# Supplementary material for: Cumulative learning enables convolutional neural network representations for small mass spectrometry data classification
Source: Nat Commun. 2020 Nov 5;11:5595. doi: 10.1038/s41467-020-19354-z (PMC7644674; doi:10.1038/s41467-020-19354-z)
Supplement: Supplementary file 1 — Supplementary Information [file 41467_2020_19354_MOESM1_ESM.pdf]

# Cumulative Learning Enables Convolutional Neural Network Representations for Small Mass Spectrometry Data Classification

Khawla Seddiki[1,2], Philippe Saudemont[2], Frédéric Precioso[3], Nina Ogrinc[2], Maxence Wisztorski[2], Michel Salzet[2], Isabelle Fournier[2*], Arnaud Droit[1*]

1 Centre de Recherche du CHU de Québec - Université Laval, Québec City, QC, Canada
2 Univ. Lille, Inserm, CHU Lille, U1192-Protéomique Réponse Inflammatoire Spectrométrie de Masse-PRISM, F-59000 Lille, France
3 Université Côte d'Azur, CNRS, INRIA, I3S, Sophia Antipolis, France

* Correspondence : `isabelle.fournier@univ-lille.fr and arnaud.droit@crchudequebec.ulaval.ca`

## Neural Architecture Design

In order to design an adequate neural architecture for a new problem, the complexity of the neural network model should be related to the complexity of both the task and the data space. However despite recent advances, the current knowledge on the capacity of a neural network, i.e. the complexity of expressible solutions, and the understanding on the relation between this capacity and the problem complexity remains limited and lead to only very broad conclusions. For instance in[1], Friedland et al. present engineering principles to quantify the capabilities of a neural network given its size and architecture as memory capacity, which allows the comparison of the efficiency of different architectures independently of a task. They conclude on the benefit of over-parameterizing (a.k.a. over-specifying) the network over its generalization power that has been further investigated by Neyshabur et al.[2] and Livni et al.[3]. In this latter work, the authors even propose a new complexity measure for two layer RelU networks leading them to present a matching lower bound for the Rademacher complexity which improves over previous capacity lower bounds for neural networks[4]. Regularization also plays an important role since, for instance, it has been demonstrated both theoretically and empirically that dropout regularization is related to a smaller Rademacher complexity for the given neural network. Dropout regularization is even able to reduce exponentially Rademacher complexity for deep networks[5]. All these very interesting works, and many other recent ones, can provide good practices (that we will follow in the next sections) to design a network for a new problem but not really a direct recipe to build it.

The highest trend currently to design (deep) neural networks for a new problem, is to rely on Neural Architecture Search (NAS)[6], that can be seen as subfield of AutoML and is also related to hyper-parameter optimization[7]. Several techniques have been proposed and receive a high attention: AdaNet[8], PNAS[9], REINFORCE[10], DARTS[11], to name a few. These different strategies cover ensemble learning, Bayesian Optimisation, Reinforcement Learning, gradient-based, Random Search or Neuro-Evolution. They aim to define the neural network architecture hyper-parameters: number of layers, number of neurons on each layer, kind of layers, etc. However all these techniques have the same requirement: enough training data for the exploration of architectures in the search space to be meaningful. Indeed, in all these works, the main evaluation protocol is to start from a small training set, CIFAR-10, to train a small architecture then to incrementally increase the architecture along with the training set until reaching state-of-the-art performances on the biggest training set, ImageNet. CIFAR-10 is an image dataset of 60.000 samples (50.000 training samples) for 10 classes and each sample is of size 32x32=1024. As a comparison, the biggest mass spectra dataset that we consider in this work is the rat brain dataset (detailed in section Datasets) of 10.100 samples for 2 classes and each sample is of size 7.084, 15.000 or even 19.000 depending on the experiment. There is no intuitive reason to consider that the patterns to be learnt for discriminating mass spectrum data classes are less complex than the ones to discriminate the CIFAR-10 classes, thus we can expect that a reasonably similar amount of training data would be required to start benefiting from NAS strategies. However, our training samples are 6 times fewer than CIFAR-10 ones (number of spectra) while being at the same time 20 times larger (number of features). This is the worst case scenario to apply any of the NAS strategies.

When the training set is too small to be confident in the search process, the standard strategy is *Transfer Learning*, which relies on existing architectures developed for similar data and similar problems. A survey on transfer learning approaches[12] describes very well the different scenarii or settings of transfer.

Let us now rephrase definitions and notations from[12] relevant to our subsequent description of *Inductive Transfer Learning.* First we recall the notations: a domain is a pair $D = \{X, P(X)\}$, and a task is defined as a pair $T = \{Y, P(Y|X)\}$.

**Definition 1** (Transfer Learning): Given a source domain $D_S$ and learning task $T_S$, a target domain $D_T$ and learning task $T_T$ , *Transfer Learning* aims to help improve the learning of the target predictive function $f_T()$ in $D_T$ using the knowledge in $D_S$ and $T_S$, where $D_S \neq D_T$, or $T_S \neq T_T$.

In the previous definition, the condition $D_S \neq D_T$ implies that either $X_S \neq X_T$ or $P_S(X) \neq P_T(X)$, and the condition $T_S \neq T_T$ implies that either $Y_S \neq Y_T$ or $P(Y_S|X_S) \neq P(Y_T|X_T)$.

**Definition 2** (Inductive Transfer Learning) Given a source domain $D_S$ and a learning task $T_S$, a target domain $D_T$ and a learning task $T_T$, *Inductive Transfer Learning* aims at helping improving the learning of the target predictive function $f_T()$ in $D_T$ using the knowledge in $D_S$ and $T_S$, where $T_S \neq T_T$.

In this setting, some labeled data (even only few data) in the target domain are required to *induce* an objective predictive model $f_T()$ for use in the target domain. For the sake of simplicity, from here we will omit *inductive* term to keep only *Transfer Learning.*

In this work, we explore the potential and limitations of *Transfer Learning* setting for MS data classification and show that the framework that we call *Cumulative Learning* is able to generalize transfer learning principles in extreme cases of very few training data. To this end, we investigate several models as candidates for predictive source model $f_S(.)$, to transfer the knowledge from $\{D_S, T_S\}$ to $\{D_T, T_T\}$.

## Methodology overview

We provide a general overview of our proposed transfer and cumulative learning procedure prior to going more in depth in the detailed protocol (section Experimental design). As shown in Figure 1, transfer learning makes a single knowledge transfer from $\{D_S, T_S\}$ to $\{D_T, T_T\}$, while the cumulative learning makes use of a knowledge transfer processes through several source domains and source tasks to improve the prediction performance of the model in the target domain for the target task. In Figure 1, ($D_{S_1}$, $T_{S_1}$),..., ($D_{S_n}$, $T_{S_n}$) represent source domains and corresponding learning tasks respectively. ($D_T$, $T_T$) represents the target domain and corresponding learning task. $f_T$ denotes the classifier that is obtained by training transfer learning or cumulative learning using the dataset in target and source domain.

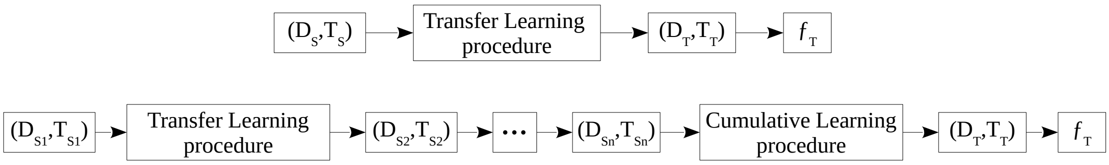


**Supplementary Fig. 1** : Framework of Transfer Learning and Cumulative Learning. Domain is labeled as D and Task is labeled as T.

Because the cumulative learning is an unexplored concept and no research has been undertaken on it yet, we propose two approaches to explore its properties :

- CNN model is first trained with the source domain dataset ($D_{S_1}$, $T_{S_1}$), then with a second source domain dataset ($D_{S_2}$, $T_{S_2}$), and finally with the target domain dataset ($D_T$, $T_T$).

- CNN model is first trained on the first source domain dataset ($D_{S_1}$, $T_{S_1}$), then with a second ($D_{S_2}$, $T_{S_2}$), and a third source domain dataset ($D_{S_3}$, $T_{S_3}$) before training it finally with the target domain dataset ($D_T$, $T_T$).

We do not consider learning the predictive model simultaneously on different domains as it is the case in multi-task learning[12], but we rather learn a predictive model on one domain to transfer the knowledge to the next domain sequentially, and so on.

## Source model candidates

In order to apply transfer learning, we first have to determine a relevant architecture on which to build our source model. In this work, we are interested in identifying patterns in 1D signal of MS spectra. Since there is no sequential organization of molecules in the spectra (two successive peaks on the mass/charge (m/z) axis are members of the same ion series but are not necessarily biologically dependent on each other), we do not consider Recurrent Neural Networks (RNNs). We are looking for identifying spatial static patterns which allow us to discriminate spectra from different classes. The adequate neural architecture in this case are CNNs.

Only few studies of input signal classification or regression using 1D-CNNs have been described in the literature, for vibrational spectroscopy data[13], for Near-Infrared (NIR) spectroscopy data[14–16] or for Raman spectroscopy data[17]. Acquarelli et al.[13] use a shallow CNN without fully-connected layers. Zhang et al.[14] design a DeepSpectra model composed of three convolutional layers, one flatten layer, and one fully connected layer. Ni et al.[15] propose a VWCNN model composed of important factor blocks, three convolutional layers, and a multi-layer perceptron composed of three fully connected layers. Malek et al.[16] propose a regression architecture based on an alternation of convolutional layers and subsampling layers. Finally Liu et al.[17] design a variant of the CNN, *LeNet*, comprising three convolutional layers and two fully-connected layers. All these studies applied 1D-CNN models to spectra analysis in the chemometric domain. We have found no description of 1D-CNN used in conjunction with MS data. Although the chemometric data used in the publication cited before is a 1D signal, our MS signal is distinguished by its shape which is relatively more complex. In addition, analyzing biological samples with MS results in a significant high signal variability due to differences in peak heights (peak intensities vary from cell-to-cell) and the fact that all peaks do not show up in each sample (peak presences/absences). This variability results mainly in peak translations (shifts) and intensities variability from one spectrum to another. That is where CNNs robustness or invariance to spatial transformation properties handle the inter-class variability. Figure 2 illustrates the typical within-class variance of three spectra in the Myxosarcoma tissue type.

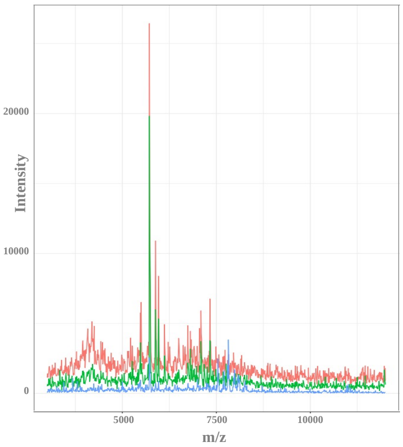


**Supplementary Fig. 2** : An example of three spectra from canine Myxosarcoma type indicating the within-class variation.

Our starting point in this study is to benefit from the existing spectral models and from successful 2D architectures developed for image analysis. The first of these is variant_Lecun adapted from[18], the second is variant_LeNet developed by[17], and the third is variant_VGG9 adapted from[19]. Variant_Lecun (model 1) contains two convolutional layers and two fully connected layers. Variant_LeNet (model 2) includes three convolutional layers and two fully connected layers. Variant_VGG9 (model 3) is the deepest, with six convolutional layers and three fully connected layers. CNNs architectures and their hyper-parameters are shown in Figure 3.

Following neural architecture evolution in the field of 2D images classification and the heuristic of over-parameterizing (or over-specifying) the network (Neural Architecture Design section), deeper 1D-CNN architectures such as VGG16 and VGG19 are also evaluated on the canine sarcoma dataset, but produce worse classification results (data not shown) most probably because these models are too big for the available training samples which are both too few and too large[3].

## Source model optimization

Once we have identified candidates as predictive source models $f_S(.)$ we have optimized them on our data and for our problem. We present in this section all the hyper-parameters we have analyzed.

We begin with an investigation of various convolutional filter sizes for the extraction of spectral features. We notice that large filter sizes are more effective than those optimized for 2D images (pixel features detected with filter sizes of 3x3). Furthermore, unlike images where the same filter size is used in all the convolutional layers, spectral patterns are optimally extracted with filters of different sizes at each layer. All this indicates that features extracted from spectral data are more complex than those seen in images. Our kernel sizes are

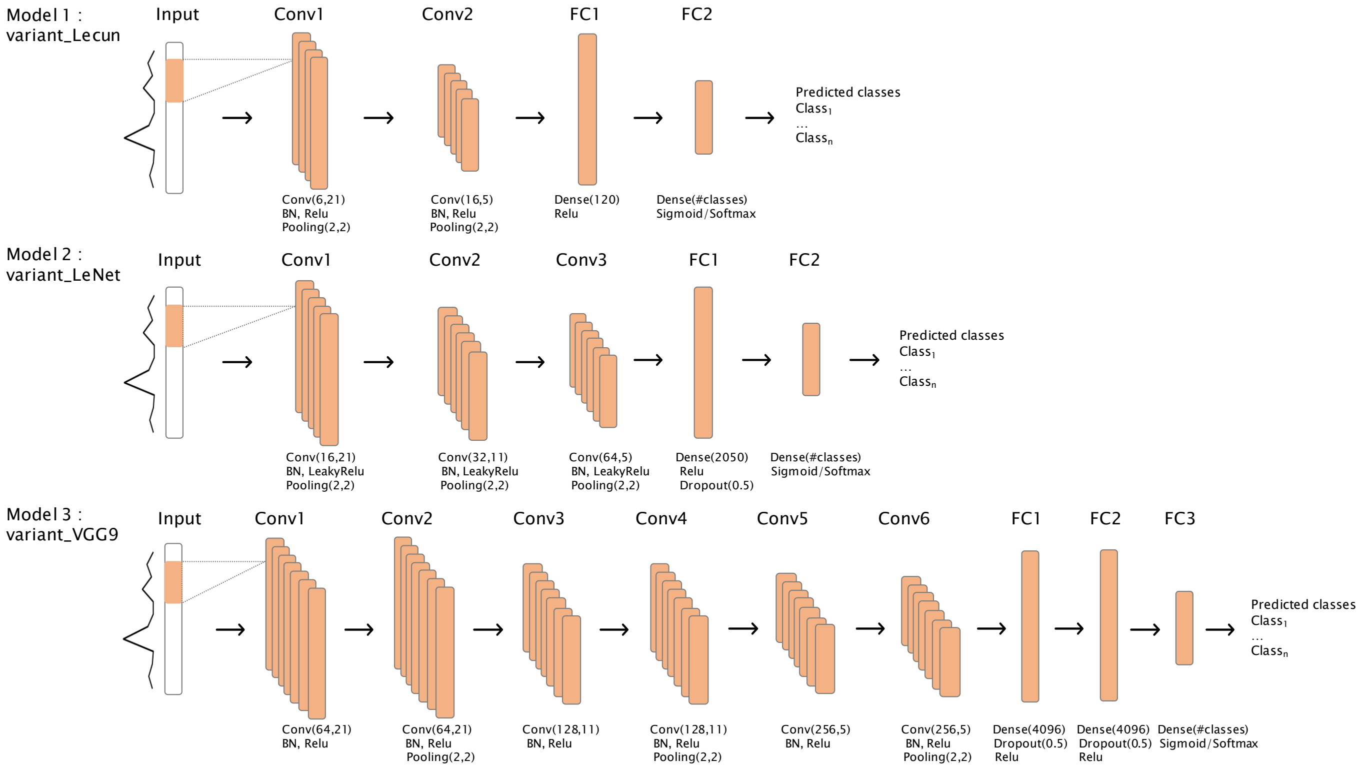


**Supplementary Fig. 3** : Architectures of the three CNN models. Convolutional layers are labeled as Conv, flatten layer as Flatten, fully connected layers as FC, Batch normalization as BN. Conv(6,21) means 6 kernels with size 21. Pooling(2,2) means pool size of 2 with a stride of 2.

both large enough to cover a small subset of peaks in the samples so that the model does not need many layers and avoid over-fitting, and not too large so that no detailed information is lost due to smoothing effects. The traditional approach to implement convolutional filters is to impose a small filter size at the lower layers and increase the filter size progressively across layers to identify salient higher level non-linearities[20]. We implement this approach as well as its reverse by allowing for greater non-linearity at the lower level and then constraining sizes in the higher levels. We find that allocating large filters at the low level and then shrinking them in the higher levels improves our models performance.

The regularizer technique, the optimizer algorithm, and the learning rate reveal significant effects on classification accuracy. Batch normalization, used after each convolutional layer to avoid over-fitting, is found superior to the dropout technique and $l_1/l_2$ regularization. The Adam optimizer provides better results than the standard Stochastic Gradient Descent (SGD) algorithm. Adam is associated to a cross-entropy loss function. We also find that Max-Pooling is very important in order to account for peak shift invariance along the m/z dimension. The results of this in-depth analysis and the resulting numerical values found for all hyper-parameters for our experiments are detailed in the Hyper-parameter optimization section.

## Hyper-parameter optimization

We evaluate the effects of hyper-parameter value alterations on the classification accuracy of the clinical datasets by CNNs. After identifying the architectures of the source candidate models, the first parameter to be analyzed is the CNN filter sizes. For the variant_LeNet model we use the same hyper-parameters as described in[17]. For variant_Lecun and variant_VGG9 models, we begin our investigation by testing the CNNs hyper-parameters optimized for images. We find that filter sizes adjusted to extract images features (3x3) are not adapted to extract spectra patterns. We subsequently expand them gradually until we find that 21 at the first convolutional layer and 5 at the last convolutional are the best ones. We settle on the same number of filters as it performs optimally. This investigation is followed by a search of various learning rates, including 0.1, 0.01, and 0.001. We reduce the learning rate (division by 10) when the validation set accuracy stops improving during 10 epochs. The *early stopping* is set after 20 epochs without the validation set accuracy improvement. No big discrepancies are noticed in the learning curves except for canine binary classification task because of the large class imbalance. We therefore reduce the patience epochs parameter from 20 to 5 for this specific task to avoid over-fitting. We also investigate the use of two optimizer algorithms,

including Adam and Stochastic Gradient Descent (SGD). The Adam optimizer with default hyper-parameters $\beta_1 = 0.9$, $\beta_2 = 0.999$ and a constant learning rate of $\eta = 0.001$ is found superior to the SGD algorithm. We search the use of various batch sizes, including 64, 128, and 256. This evaluation is also done in terms of regularizer technique by adding either batch normalization, dropout of 0.1, 0.5 or $l_1/l_2$ regularization after each convolutional layer. Batch normalization is found superior to the dropout technique and $l_1/l_2$ regularization. Since batch size does not affect the results, it is set at 256. ReLu (models 1 and 3) and Leaky Relu (model 2) are chosen as the activation function for each convolutional layer.

## Matrix construction

Raw SpiderMass spectra are converted into mzXML format using the 64-bit MSConvert tool (version 3.0), part of the ProteoWizard suite[21]. Spectra with a total ion count (TIC) exceeding $1.e^4$ count for irradiation detection are selected using the MSnbase package (version 1.20.7, R version 3.4.4)[22]. Raw ovarian datasets are imported into a csv file format. In this study, we focus on lipids and metabolites as the main species observed in the 100-2000 m/z range with the SpiderMass. Multiple studies have shed light on the role of lipid metabolism deregulation in cancer development[23–25]. Some tumors exhibit a lipogenic phenotype, since membrane lipids are synthesized rapidly and with high turnover in malignant cells[26,27], suggesting the relevance of a lipid-based classification model. Recent microbial taxonomy studies have also demonstrated the possibility of biotyping pathogens using their lipid composition[28]. The classification models obtained using SpiderMass datasets are therefore based on lipid profiles. To allow a fair comparison with the original paper, mass spectra acquired from public ovarian datasets (high and low-resolution) are restricted to the m/z range from 700 to 12.000[29]. The classification models obtained using public ovarian datasets are therefore based on lipids and proteins patterns.

All datasets used for the transfer and cumulative learning must have the same size because the neural network has a fixed number of inputs, so vectors fed to the neural network must have the same size. We choose the binning as a way to equalize data size. The MALDI rat brain dataset is binned in three different ways depending on whether it has been used for transfer or cumulative learning. The bigger the bin size, the more information will be summarized in the resulting intensity matrix. Typically in the MALDI-TOF datasets, peak width increases with increasing m/z values[30]. This peak broadening in the rat brain dataset requires to use larger bin size (0.1 or 0.2) to take into account this broadening, in addition to other instrument variation. In the following Table 1 we compute the classification accuracies of the rat brain dataset to assess the effect of the different binning sizes on the CNN classification performances.

| # features | Model 1 | Model 2 | Model 3 |
|---|---|---|---|
| Without binning (38.500 features) | 0.995 ± 0.001 | 0.995 ± 0.001 | 0.994 ± 0.002 |
| Binning at 19.000 features | 0.996 ± 0.002 | 0.994 ± 0.002 | 0.997 ± 0.000 |
| Binning at 15.000 features | 0.996 ± 0.000 | 0.990 ± 0.001 | 0.994 ± 0.001 |
| Binning at 7084 features | 0.989 ± 0.002 | 0.990 ± 0.002 | 0.992 ± 0.001 |

**Supplementary Table 1** : Overall accuracies of the rat brain dataset with three CNN architectures. Each task is expressed in accuracy ± standard variation over 10 independent iterations.

As shown in the results, the performance of the three CNNs models does not decrease significantly with the different binning size. While binning summarizes spectral information, data representation is preserved and is efficiently transferred to the other datasets. Nevertheless, binning at 7084 features requires a longer training time (60 epochs) and a reduction of the learning rate (division by 0.1 every 10 epochs) when the validation set accuracy stopped improving. Our future research will benefit from all the advances in understanding the effect of compression on the extracted data representation.

## Data exploration

There is no special requirement on the type of the source domain dataset, any dataset could be used but the bigger the better. The notion of "enough data" varies depending on the data properties (level of resolution, number of classes, presence or not of unbalanced classes, class ambiguities, amount of samples, dimension of each sample, etc.). Unless exploring different architectures on the data by many test-error iterations, it is hard to determine in advance what is the lowest number of spectra one should have or if the number of available samples is enough to get a performing model. We detail below some elements regarding the data adaptation:

### Varying the number of samples

We use the MALDI rat brain dataset as the source domain because it contains the largest number of spectra. The rat brain dataset size is considerably small compared to the ImageNet database for instance (more than 14 million of images), used in most of the transfer learning examples in the literature[31]. Our hypothesis, when the transfer learning from the rat brain dataset is not efficient enough as in the case of data heterogeneity (canine sarcoma dataset) and low-resolution (ovarian dataset), is that the unsatisfactory classification performance may be due to the fact that the source domain data is not large enough to learn an efficient data representation. We could have varied the number of rat brain spectra used for the transfer (e.g. 1000 spectra, 5000 spectra, etc.) but because 10.100 spectra are not enough to learn an efficient data representation it is not useful to further reduce the number of spectra.

### Varying the bin size

The choice of the binning size depends on the nature of the mass analyzer and the information obtained. To allow a fair comparison with the original paper, public ovarian datasets (high-resolution and low-resolution) are binned as described in the original paper regarding the MS instrument specificities[29]. For the SpiderMass datasets, binning at 0.01 is not tested because it is very expensive in terms of the needed computing resources (71 millions $\sim$ 24 billions parameters) as shown in Table 2.

| Bin size | Datasets | Model 1 | Model 2 | Model 3 |
|---|---|---|---|---|
| Bin=1 | Canine sarcoma | 717.557 | 23.615.874 | 199.047.362 |
| | Microorganisms | 909.557 | 30.169.474 | 251.476.162 |
| Bin=0.1 | Canine sarcoma | 7.197.557 | 244.865.410 | 1.969.043.650 |
| | Microorganisms | 9.117.557 | 310.401.410 | 2.493.331.650 |
| Bin=0.01 | Canine sarcoma | 71.997.557 | 2.456.705.410 | 19.663.763.650 |
| | Microorganisms | 91.197.557 | 3.112.065.410 | 24.906.643.650 |

**Supplementary Table 2** : Number of the three CNN architectures parameters to estimate for canine sarcoma and microorganism datasets

SpiderMass spectra are binned to 0.1 Da for the subsequent analyses.

## CNN architectures performances

| Model 1 | | Model 2 | | Model 3 | |
|---|---|---|---|---|---|
| Accuracy = 0.98 ± 0.00 | | Accuracy = 0.96 ± 0.01 | | Accuracy = 0.96 ± 0.01 | |
| **Sensitivity** | **Specificity** | **Sensitivity** | **Specificity** | **Sensitivity** | **Specificity** |
| 0.979 | 0.997 | 0.958 | 0.996 | 0.936 | 0.991 |

**Supplementary Table 3** : Overall accuracy, sensitivity, and specificity values for 2-class canine sarcoma classification

| **Model 1** | | Predicted 0 | Predicted 1 |
|---|---|---|---|
| Actual | 0 | 96 | 1 |
| Actual | 1 | 2 | 343 |

| **Model 2** | | Predicted 0 | Predicted 1 |
|---|---|---|---|
| Actual | 0 | 92 | 1 |
| Actual | 1 | 4 | 326 |

| **Model 3** | | Predicted 0 | Predicted 1 |
|---|---|---|---|
| Actual | 0 | 88 | 3 |
| Actual | 1 | 6 | 344 |

**Supplementary Table 4** : Confusion matrix for 2-class canine sarcoma classification

| | Model 1 | | Model 2 | | Model 3 | |
|---|---|---|---|---|---|---|
| | Accuracy = 0.88 ± 0.03 | | Accuracy = 0.88 ± 0.02 | | Accuracy = 0.90 ± 0.01 | |
| **Index** | **Sensitivity** | **Specificity** | **Sensitivity** | **Specificity** | **Sensitivity** | **Specificity** |
| 0 | 1.000 | 0.985 | 0.961 | 0.994 | 0.978 | 0.990 |
| 1 | 0.714 | 1.000 | 0.916 | 1.000 | 0.900 | 1.000 |
| 2 | 0.843 | 0.977 | 0.873 | 0.961 | 0.855 | 0.985 |
| 3 | 0.838 | 0.992 | 0.913 | 0.986 | 0.777 | 0.997 |
| 4 | 0.666 | 0.995 | 0.937 | 1.000 | 1.000 | 0.994 |
| 5 | 0.883 | 0.976 | 0.791 | 0.968 | 0.886 | 0.965 |
| 6 | 0.925 | 0.966 | 0.913 | 0.955 | 0.916 | 0.965 |
| 7 | 0.888 | 0.997 | 0.900 | 0.997 | 0.923 | 0.992 |
| 8 | 0.777 | 0.995 | 0.866 | 1.000 | 0.833 | 1.000 |
| 9 | 0.956 | 0.990 | 0.833 | 1.000 | 0.941 | 1.000 |
| 10 | 0.800 | 1.000 | 0.812 | 1.000 | 0.833 | 1.000 |
| 11 | 0.785 | 1.000 | 0.727 | 1.000 | 0.769 | 0.994 |

**Supplementary Table 5** : Overall accuracy, sensitivity, and specificity values for 12-class canine sarcoma classification

**Model 1** (Predicted)

| Actual | 0 | 1 | 2 | 3 | 4 | 5 | 6 | 7 | 8 | 9 | 10 | 11 |
|---|---|---|---|---|---|---|---|---|---|---|---|---|
| 0 | 91 | 2 | 1 | 0 | 0 | 0 | 2 | 0 | 0 | 0 | 0 | 0 |
| 1 | 0 | 8 | 0 | 0 | 0 | 0 | 0 | 0 | 0 | 0 | 0 | 0 |
| 2 | 0 | 0 | 70 | 3 | 1 | 3 | 0 | 0 | 0 | 0 | 0 | 1 |
| 3 | 0 | 0 | 2 | 26 | 0 | 1 | 0 | 0 | 0 | 0 | 0 | 0 |
| 4 | 0 | 0 | 2 | 0 | 4 | 0 | 0 | 0 | 0 | 0 | 0 | 0 |
| 5 | 0 | 0 | 4 | 2 | 1 | 53 | 1 | 0 | 0 | 0 | 0 | 1 |
| 6 | 0 | 0 | 3 | 0 | 0 | 3 | 74 | 2 | 1 | 1 | 1 | 1 |
| 7 | 0 | 0 | 1 | 0 | 0 | 0 | 0 | 16 | 0 | 0 | 0 | 0 |
| 8 | 0 | 0 | 0 | 0 | 0 | 0 | 2 | 0 | 7 | 0 | 0 | 0 |
| 9 | 0 | 0 | 0 | 0 | 0 | 0 | 1 | 0 | 1 | 22 | 2 | 0 |
| 10 | 0 | 0 | 0 | 0 | 0 | 0 | 0 | 0 | 0 | 0 | 12 | 0 |
| 11 | 0 | 0 | 0 | 0 | 0 | 0 | 0 | 0 | 0 | 0 | 0 | 11 |

**Model 2** (Predicted)

| Actual | 0 | 1 | 2 | 3 | 4 | 5 | 6 | 7 | 8 | 9 | 10 | 11 |
|---|---|---|---|---|---|---|---|---|---|---|---|---|
| 0 | 99 | 0 | 0 | 0 | 0 | 1 | 0 | 1 | 0 | 0 | 0 | 0 |
| 1 | 0 | 11 | 0 | 0 | 0 | 0 | 0 | 0 | 0 | 0 | 0 | 0 |
| 2 | 0 | 0 | 76 | 1 | 0 | 7 | 1 | 0 | 2 | 2 | 1 | 0 |
| 3 | 0 | 0 | 2 | 21 | 1 | 1 | 1 | 0 | 0 | 0 | 0 | 1 |
| 4 | 0 | 0 | 0 | 0 | 15 | 0 | 0 | 0 | 0 | 0 | 0 | 0 |
| 5 | 0 | 0 | 5 | 1 | 0 | 53 | 4 | 0 | 0 | 0 | 0 | 2 |
| 6 | 3 | 1 | 4 | 0 | 0 | 5 | 63 | 0 | 0 | 2 | 2 | 0 |
| 7 | 1 | 0 | 0 | 0 | 0 | 0 | 0 | 9 | 0 | 0 | 0 | 0 |
| 8 | 0 | 0 | 0 | 0 | 0 | 0 | 0 | 0 | 13 | 0 | 0 | 0 |
| 9 | 0 | 0 | 0 | 0 | 0 | 0 | 0 | 0 | 0 | 20 | 0 | 0 |
| 10 | 0 | 0 | 0 | 0 | 0 | 0 | 0 | 0 | 0 | 0 | 13 | 0 |
| 11 | 0 | 0 | 0 | 0 | 0 | 0 | 0 | 0 | 0 | 0 | 0 | 8 |

**Model 3** (Predicted)

| Actual | 0 | 1 | 2 | 3 | 4 | 5 | 6 | 7 | 8 | 9 | 10 | 11 |
|---|---|---|---|---|---|---|---|---|---|---|---|---|
| 0 | 90 | 0 | 0 | 0 | 0 | 0 | 3 | 0 | 0 | 0 | 0 | 0 |
| 1 | 0 | 9 | 0 | 0 | 0 | 0 | 0 | 0 | 0 | 0 | 0 | 0 |
| 2 | 0 | 1 | 59 | 1 | 0 | 1 | 0 | 0 | 0 | 0 | 2 | 0 |
| 3 | 0 | 0 | 1 | 14 | 0 | 0 | 0 | 0 | 0 | 0 | 0 | 0 |
| 4 | 0 | 0 | 1 | 0 | 9 | 1 | 0 | 0 | 0 | 0 | 0 | 0 |
| 5 | 0 | 0 | 5 | 2 | 0 | 47 | 2 | 0 | 0 | 0 | 0 | 3 |
| 6 | 2 | 0 | 3 | 1 | 0 | 2 | 77 | 1 | 1 | 1 | 0 | 0 |
| 7 | 0 | 0 | 0 | 0 | 0 | 1 | 2 | 12 | 0 | 0 | 0 | 0 |
| 8 | 0 | 0 | 0 | 0 | 0 | 0 | 0 | 0 | 10 | 0 | 0 | 0 |
| 9 | 0 | 0 | 0 | 0 | 0 | 0 | 0 | 0 | 0 | 16 | 0 | 0 |
| 10 | 0 | 0 | 0 | 0 | 0 | 0 | 0 | 0 | 0 | 0 | 10 | 0 |
| 11 | 0 | 0 | 0 | 0 | 0 | 1 | 0 | 0 | 1 | 0 | 0 | 10 |

**Supplementary Table 6** : Confusion matrix for 12-class canine sarcoma classification

| | Model 1 | | Model 2 | | Model 3 | |
|---|---|---|---|---|---|---|
| | Accuracy = 0.91 ± 0.03 | | Accuracy = 0.52 ± 0.11 | | Accuracy = 0.67 ± 0.09 | |
| **Index** | **Sensitivity** | **Specificity** | **Sensitivity** | **Specificity** | **Sensitivity** | **Specificity** |
| 0 | 1.000 | 0.913 | 1.000 | 0.200 | 1.000 | 0.750 |
| 1 | 0.866 | 1.000 | 0.000 | 1.000 | 0.000 | 1.000 |
| 2 | 1.000 | 1.000 | 1.000 | 1.000 | 1.000 | 0.761 |

**Supplementary Table 7** : Overall accuracy, sensitivity, and specificity values for 3-class microorganisms classification

**Model 1** (Predicted)

| Actual | 0 | 1 | 2 |
|---|---|---|---|
| 0 | 5 | 2 | 0 |
| 1 | 0 | 11 | 0 |
| 2 | 0 | 11 | 0 |

**Model 2** (Predicted)

| Actual | 0 | 1 | 2 |
|---|---|---|---|
| 0 | 10 | 8 | 0 |
| 1 | 0 | 0 | 0 |
| 2 | 0 | 0 | 2 |

**Model 3** (Predicted)

| Actual | 0 | 1 | 2 |
|---|---|---|---|
| 0 | 12 | 4 | 0 |
| 1 | 0 | 0 | 0 |
| 2 | 0 | 5 | 7 |

**Supplementary Table 8** : Confusion matrix for 3-class microorganisms classification

| | Model 1 | | Model 2 | | Model 3 | |
|---|---|---|---|---|---|---|
| | Accuracy = 0.89 ± 0.02 | | Accuracy = 0.68 ± 0.03 | | Accuracy = 0.61 ± 0.13 | |
| **Index** | **Sensitivity** | **Specificity** | **Sensitivity** | **Specificity** | **Sensitivity** | **Specificity** |
| 0 | 1.000 | 1.000 | 1.000 | 1.000 | 0.000 | 1.000 |
| 1 | 0.000 | 1.000 | 0.000 | 1.000 | 0.428 | 1.000 |
| 2 | 1.000 | 0.678 | 1.000 | 0.673 | 1.000 | 1.000 |
| 3 | 0.000 | 1.000 | 0.000 | 1.000 | 1.000 | 1.000 |
| 4 | 1.000 | 0.954 | 1.000 | 0.959 | 1.000 | 0.555 |

**Supplementary Table 9** : Overall accuracy, sensitivity, and specificity values for 5-class microorganisms classification

**Model 1** (Predicted)

| Actual | 0 | 1 | 2 | 3 | 4 |
|---|---|---|---|---|---|
| 0 | 8 | 0 | 0 | 0 | 0 |
| 1 | 0 | 0 | 0 | 0 | 0 |
| 2 | 0 | 7 | 4 | 2 | 0 |
| 3 | 0 | 0 | 0 | 0 | 0 |
| 4 | 0 | 1 | 0 | 0 | 10 |

**Model 2** (Predicted)

| Actual | 0 | 1 | 2 | 3 | 4 |
|---|---|---|---|---|---|
| 0 | 8 | 0 | 0 | 0 | 0 |
| 1 | 0 | 0 | 0 | 0 | 0 |
| 2 | 0 | 7 | 4 | 2 | 0 |
| 3 | 0 | 0 | 0 | 0 | 0 |
| 4 | 0 | 1 | 0 | 0 | 10 |

**Model 3** (Predicted)

| Actual | 0 | 1 | 2 | 3 | 4 |
|---|---|---|---|---|---|
| 0 | 0 | 0 | 0 | 0 | 0 |
| 1 | 0 | 3 | 0 | 0 | 0 |
| 2 | 0 | 0 | 3 | 0 | 0 |
| 3 | 0 | 0 | 0 | 4 | 0 |
| 4 | 4 | 4 | 0 | 0 | 3 |

**Supplementary Table 10** : Confusion matrix for 5-class microorganisms classification

## Transfer learning performances

| | Model 1 | | Model 2 | | Model 3 | |
|---|---|---|---|---|---|---|
| | Accuracy = 0.90 ± 0.01 | | Accuracy = 0.92 ± 0.01 | | Accuracy = 0.93 ± 0.02 | |
| **Index** | **Sensitivity** | **Specificity** | **Sensitivity** | **Specificity** | **Sensitivity** | **Specificity** |
| 0 | 0.889 | 1.000 | 0.730 | 1.000 | 0.946 | 1.000 |
| 1 | 1.000 | 1.000 | 1.000 | 1.000 | 1.000 | 1.000 |
| 2 | 0.942 | 0.983 | 0.988 | 1.000 | 0.952 | 0.989 |
| 3 | 0.813 | 0.996 | 1.000 | 0.997 | 0.956 | 0.996 |
| 4 | 0.882 | 1.000 | 1.000 | 1.000 | 0.875 | 1.000 |
| 5 | 0.950 | 0.977 | 0.985 | 0.997 | 0.925 | 0.986 |
| 6 | 0.947 | 0.944 | 1.000 | 0.919 | 0.879 | 0.982 |
| 7 | 1.000 | 0.998 | 1.000 | 1.000 | 1.000 | 1.000 |
| 8 | 0.823 | 1.000 | 0.818 | 1.000 | 0.900 | 0.997 |
| 9 | 0.757 | 0.992 | 0.958 | 0.997 | 1.000 | 0.972 |
| 10 | 1.000 | 1.000 | 1.000 | 1.000 | 0.818 | 1.000 |
| 11 | 1.000 | 1.000 | 1.000 | 1.000 | 0.909 | 0.997 |

**Supplementary Table 11** : Overall accuracy, sensitivity, and specificity values for 12-class canine sarcoma classification

**Model 1** (Predicted / Actual)

| Actual \ Predicted | 0 | 1 | 2 | 3 | 4 | 5 | 6 | 7 | 8 | 9 | 10 | 11 |
|---|---|---|---|---|---|---|---|---|---|---|---|---|
| 0 | 82 | 0 | 0 | 0 | 0 | 0 | 0 | 0 | 0 | 0 | 0 | 0 |
| 1 | 0 | 19 | 0 | 0 | 0 | 0 | 0 | 0 | 0 | 0 | 0 | 0 |
| 2 | 0 | 0 | 65 | 4 | 0 | 4 | 0 | 0 | 0 | 0 | 1 | 0 |
| 3 | 0 | 0 | 1 | 35 | 1 | 0 | 0 | 0 | 0 | 0 | 0 | 0 |
| 4 | 0 | 0 | 0 | 0 | 15 | 0 | 0 | 0 | 0 | 0 | 0 | 0 |
| 5 | 0 | 0 | 4 | 3 | 1 | 95 | 5 | 0 | 0 | 0 | 0 | 0 |
| 6 | 15 | 0 | 2 | 1 | 0 | 1 | 80 | 0 | 1 | 8 | 2 | 1 |
| 7 | 0 | 0 | 0 | 0 | 0 | 0 | 1 | 18 | 0 | 0 | 0 | 0 |
| 8 | 0 | 0 | 0 | 0 | 0 | 0 | 0 | 0 | 14 | 0 | 0 | 0 |
| 9 | 1 | 0 | 0 | 0 | 0 | 0 | 0 | 0 | 2 | 25 | 2 | 0 |
| 10 | 0 | 0 | 0 | 0 | 0 | 0 | 0 | 0 | 0 | 0 | 16 | 0 |
| 11 | 0 | 0 | 0 | 0 | 0 | 0 | 0 | 0 | 0 | 0 | 0 | 20 |

**Model 2** (Predicted / Actual)

| Actual \ Predicted | 0 | 1 | 2 | 3 | 4 | 5 | 6 | 7 | 8 | 9 | 10 | 11 |
|---|---|---|---|---|---|---|---|---|---|---|---|---|
| 0 | 86 | 0 | 0 | 0 | 0 | 0 | 0 | 0 | 0 | 0 | 0 | 0 |
| 1 | 0 | 13 | 0 | 0 | 0 | 0 | 0 | 0 | 0 | 0 | 0 | 0 |
| 2 | 0 | 0 | 88 | 0 | 0 | 0 | 0 | 0 | 0 | 0 | 0 | 0 |
| 3 | 0 | 0 | 1 | 32 | 0 | 0 | 0 | 0 | 0 | 0 | 0 | 0 |
| 4 | 0 | 0 | 0 | 0 | 13 | 0 | 0 | 0 | 0 | 0 | 0 | 0 |
| 5 | 0 | 0 | 0 | 0 | 0 | 68 | 0 | 0 | 0 | 0 | 0 | 1 |
| 6 | 28 | 0 | 0 | 0 | 0 | 1 | 80 | 0 | 1 | 1 | 1 | 0 |
| 7 | 0 | 0 | 0 | 0 | 0 | 0 | 0 | 13 | 0 | 0 | 0 | 0 |
| 8 | 0 | 0 | 0 | 0 | 0 | 0 | 0 | 0 | 9 | 0 | 0 | 0 |
| 9 | 0 | 0 | 0 | 0 | 0 | 0 | 0 | 0 | 1 | 23 | 0 | 0 |
| 10 | 0 | 0 | 0 | 0 | 0 | 0 | 0 | 0 | 0 | 0 | 14 | 0 |
| 11 | 0 | 0 | 0 | 0 | 0 | 0 | 0 | 0 | 0 | 0 | 0 | 14 |

**Model 3** (Predicted / Actual)

| Actual \ Predicted | 0 | 1 | 2 | 3 | 4 | 5 | 6 | 7 | 8 | 9 | 10 | 11 |
|---|---|---|---|---|---|---|---|---|---|---|---|---|
| 0 | 81 | 0 | 0 | 0 | 0 | 0 | 0 | 0 | 0 | 0 | 0 | 0 |
| 1 | 0 | 10 | 0 | 0 | 0 | 0 | 0 | 0 | 0 | 0 | 0 | 0 |
| 2 | 0 | 0 | 60 | 0 | 0 | 2 | 1 | 0 | 0 | 0 | 0 | 0 |
| 3 | 0 | 0 | 0 | 22 | 0 | 1 | 0 | 0 | 0 | 0 | 0 | 0 |
| 4 | 0 | 0 | 0 | 0 | 7 | 0 | 0 | 0 | 0 | 0 | 0 | 0 |
| 5 | 0 | 0 | 0 | 1 | 1 | 50 | 2 | 0 | 0 | 0 | 0 | 0 |
| 6 | 2 | 0 | 1 | 0 | 0 | 1 | 51 | 0 | 0 | 0 | 0 | 1 |
| 7 | 0 | 0 | 0 | 0 | 0 | 0 | 0 | 10 | 0 | 0 | 0 | 0 |
| 8 | 0 | 0 | 1 | 0 | 0 | 0 | 0 | 0 | 9 | 0 | 0 | 0 |
| 9 | 1 | 0 | 1 | 0 | 0 | 0 | 4 | 0 | 1 | 17 | 2 | 0 |
| 10 | 0 | 0 | 0 | 0 | 0 | 0 | 0 | 0 | 0 | 0 | 9 | 0 |
| 11 | 1 | 0 | 0 | 0 | 0 | 0 | 0 | 0 | 0 | 0 | 0 | 12 |

**Supplementary Table 12** : Confusion matrix for 12-class canine sarcoma classification

| | Model 1 | | Model 2 | | Model 3 | |
|---|---|---|---|---|---|---|
| | Accuracy = 0.99 ± 0.00 | | Accuracy = 0.96 ± 0.01 | | Accuracy = 0.95 ± 0.02 | |
| **Index** | **Sensitivity** | **Specificity** | **Sensitivity** | **Specificity** | **Sensitivity** | **Specificity** |
| 0 | 1.000 | 1.000 | 0.900 | 1.000 | 1.000 | 0.923 |
| 1 | 1.000 | 1.000 | 1.000 | 0.937 | 0.888 | 1.000 |
| 2 | 1.000 | 1.000 | 1.000 | 1.000 | 1.000 | 1.000 |

**Supplementary Table 13** : Overall accuracy, sensitivity, and specificity values for 3-class microorganisms classification

**Model 1** (Predicted / Actual)

| Actual \ Predicted | 0 | 1 | 2 |
|---|---|---|---|
| 0 | 12 | 0 | 0 |
| 1 | 0 | 10 | 0 |
| 2 | 0 | 0 | 5 |

**Model 2** (Predicted / Actual)

| Actual \ Predicted | 0 | 1 | 2 |
|---|---|---|---|
| 0 | 9 | 0 | 0 |
| 1 | 1 | 9 | 0 |
| 2 | 0 | 0 | 6 |

**Model 3** (Predicted / Actual)

| Actual \ Predicted | 0 | 1 | 2 |
|---|---|---|---|
| 0 | 8 | 1 | 0 |
| 1 | 0 | 8 | 0 |
| 2 | 0 | 0 | 4 |

**Supplementary Table 14** : Confusion matrix for 3-class microorganisms classification

| | Model 1 | | Model 2 | | Model 3 | |
|---|---|---|---|---|---|---|
| | Accuracy = 0.99 ± 0.00 | | Accuracy = 0.99 ± 0.00 | | Accuracy = 0.96 ± 0.02 | |
| **Index** | **Sensitivity** | **Specificity** | **Sensitivity** | **Specificity** | **Sensitivity** | **Specificity** |
| 0 | 1.000 | 0.996 | 1.000 | 0.986 | 0.987 | 0.998 |
| 1 | 1.000 | 0.996 | 0.988 | 1.000 | 1.000 | 0.763 |
| 2 | 1.000 | 0.998 | 0.993 | 1.000 | 0.994 | 1.000 |
| 3 | 0.993 | 1.000 | 1.000 | 0.998 | 1.000 | 1.000 |
| 4 | 0.990 | 1.000 | 0.992 | 1.000 | 0.000 | 1.000 |

**Supplementary Table 15** : Overall accuracy, sensitivity, and specificity values for 5-class microorganisms classification

**Model 1**

| | | Predicted | | | | |
|---|---|---|---|---|---|---|
| | | 0 | 1 | 2 | 3 | 4 |
| Actual | 0 | 6 | 0 | 0 | 0 | 0 |
| | 1 | 0 | 6 | 0 | 0 | 0 |
| | 2 | 0 | 0 | 7 | 0 | 0 |
| | 3 | 0 | 0 | 0 | 5 | 0 |
| | 4 | 0 | 0 | 0 | 0 | 6 |

**Model 2**

| | | Predicted | | | | |
|---|---|---|---|---|---|---|
| | | 0 | 1 | 2 | 3 | 4 |
| Actual | 0 | 6 | 0 | 0 | 0 | 0 |
| | 1 | 0 | 6 | 0 | 0 | 0 |
| | 2 | 0 | 0 | 7 | 0 | 0 |
| | 3 | 0 | 0 | 0 | 5 | 0 |
| | 4 | 0 | 0 | 0 | 0 | 6 |

**Model 3**

| | | Predicted | | | | |
|---|---|---|---|---|---|---|
| | | 0 | 1 | 2 | 3 | 4 |
| Actual | 0 | 7 | 0 | 0 | 0 | 0 |
| | 1 | 0 | 5 | 0 | 0 | 0 |
| | 2 | 0 | 0 | 6 | 1 | 0 |
| | 3 | 0 | 0 | 0 | 4 | 0 |
| | 4 | 0 | 0 | 0 | 0 | 3 |

**Supplementary Table 16** : Confusion matrix for 5-class microorganisms classification

# Cumulative learning performances

## Scenario A

| | Model 1 | | Model 2 | | Model 3 | |
|---|---|---|---|---|---|---|
| | Accuracy = 0.92 ± 0.01 | | Accuracy = 0.95 ± 0.01 | | Accuracy = 0.94 ± 0.01 | |
| **Index** | **Sensitivity** | **Specificity** | **Sensitivity** | **Specificity** | **Sensitivity** | **Specificity** |
| 0 | 0.938 | 0.938 | 0.989 | 0.996 | 0.846 | 0.994 |
| 1 | 1.000 | 1.000 | 0.916 | 1.000 | 1.000 | 1.000 |
| 2 | 0.975 | 0.978 | 0.944 | 0.991 | 1.000 | 1.000 |
| 3 | 0.964 | 1.000 | 0.916 | 0.992 | 1.000 | 0.997 |
| 4 | 0.909 | 1.000 | 1.000 | 0.997 | 1.000 | 1.000 |
| 5 | 0.968 | 0.994 | 0.968 | 0.988 | 0.972 | 1.000 |
| 6 | 0.776 | 0.989 | 0.942 | 0.985 | 0.987 | 0.939 |
| 7 | 0.833 | 1.000 | 0.916 | 1.000 | 0.933 | 1.000 |
| 8 | 1.000 | 1.000 | 0.833 | 1.000 | 0.928 | 1.000 |
| 9 | 0.958 | 0.976 | 0.952 | 0.992 | 0.739 | 0.997 |
| 10 | 0.928 | 1.000 | 0.846 | 1.000 | 0.933 | 1.000 |
| 11 | 0.857 | 1.000 | 1.000 | 1.000 | 1.000 | 1.000 |

**Supplementary Table 17** : Overall accuracy, sensitivity, and specificity values for 12-class canine sarcoma classification

**Model 1**

| Actual \ Predicted | 0 | 1 | 2 | 3 | 4 | 5 | 6 | 7 | 8 | 9 | 10 | 11 |
|---|---|---|---|---|---|---|---|---|---|---|---|---|
| 0 | 91 | 0 | 0 | 0 | 0 | 0 | 9 | 2 | 0 | 0 | 0 | 0 |
| 1 | 0 | 13 | 0 | 0 | 0 | 0 | 0 | 0 | 0 | 0 | 0 | 0 |
| 2 | 1 | 0 | 81 | 1 | 1 | 1 | 3 | 0 | 0 | 1 | 0 | 0 |
| 3 | 0 | 0 | 0 | 27 | 0 | 0 | 0 | 0 | 0 | 0 | 0 | 0 |
| 4 | 0 | 0 | 0 | 0 | 10 | 0 | 0 | 0 | 0 | 0 | 0 | 0 |
| 5 | 0 | 0 | 1 | 0 | 0 | 62 | 1 | 0 | 0 | 0 | 0 | 0 |
| 6 | 1 | 0 | 1 | 0 | 0 | 1 | 59 | 0 | 0 | 0 | 0 | 1 |
| 7 | 0 | 0 | 0 | 0 | 0 | 0 | 0 | 10 | 0 | 0 | 0 | 0 |
| 8 | 0 | 0 | 0 | 0 | 0 | 0 | 0 | 0 | 11 | 0 | 0 | 0 |
| 9 | 4 | 0 | 0 | 0 | 0 | 0 | 4 | 0 | 0 | 23 | 1 | 1 |
| 10 | 0 | 0 | 0 | 0 | 0 | 0 | 0 | 0 | 0 | 0 | 13 | 0 |
| 11 | 0 | 0 | 0 | 0 | 0 | 0 | 0 | 0 | 0 | 0 | 0 | 12 |

**Model 2**

| Actual \ Predicted | 0 | 1 | 2 | 3 | 4 | 5 | 6 | 7 | 8 | 9 | 10 | 11 |
|---|---|---|---|---|---|---|---|---|---|---|---|---|
| 0 | 90 | 0 | 0 | 0 | 0 | 0 | 0 | 1 | 0 | 0 | 0 | 0 |
| 1 | 0 | 11 | 0 | 0 | 0 | 0 | 0 | 0 | 0 | 0 | 0 | 0 |
| 2 | 0 | 0 | 68 | 1 | 0 | 1 | 1 | 0 | 0 | 0 | 0 | 0 |
| 3 | 0 | 0 | 1 | 22 | 0 | 1 | 1 | 0 | 0 | 0 | 0 | 0 |
| 4 | 0 | 0 | 0 | 1 | 12 | 0 | 0 | 0 | 0 | 0 | 0 | 0 |
| 5 | 1 | 0 | 2 | 0 | 0 | 62 | 1 | 0 | 0 | 0 | 0 | 0 |
| 6 | 0 | 1 | 1 | 0 | 0 | 0 | 65 | 0 | 1 | 1 | 1 | 0 |
| 7 | 0 | 0 | 0 | 0 | 0 | 0 | 0 | 11 | 0 | 0 | 0 | 0 |
| 8 | 0 | 0 | 0 | 0 | 0 | 0 | 0 | 0 | 10 | 0 | 0 | 0 |
| 9 | 0 | 0 | 0 | 0 | 0 | 0 | 1 | 0 | 1 | 20 | 1 | 0 |
| 10 | 0 | 0 | 0 | 0 | 0 | 0 | 0 | 0 | 0 | 0 | 11 | 0 |
| 11 | 0 | 0 | 0 | 0 | 0 | 0 | 0 | 0 | 0 | 0 | 0 | 13 |

**Model 3**

| Actual \ Predicted | 0 | 1 | 2 | 3 | 4 | 5 | 6 | 7 | 8 | 9 | 10 | 11 |
|---|---|---|---|---|---|---|---|---|---|---|---|---|
| 0 | 88 | 0 | 0 | 0 | 0 | 0 | 1 | 1 | 0 | 0 | 0 | 0 |
| 1 | 0 | 13 | 0 | 0 | 0 | 0 | 0 | 0 | 0 | 0 | 0 | 0 |
| 2 | 0 | 0 | 85 | 0 | 0 | 0 | 0 | 0 | 0 | 0 | 0 | 0 |
| 3 | 0 | 0 | 0 | 27 | 0 | 1 | 0 | 0 | 0 | 0 | 0 | 0 |
| 4 | 0 | 0 | 0 | 0 | 14 | 0 | 0 | 0 | 0 | 0 | 0 | 0 |
| 5 | 0 | 0 | 0 | 0 | 0 | 70 | 0 | 0 | 0 | 0 | 0 | 0 |
| 6 | 16 | 0 | 0 | 0 | 0 | 1 | 80 | 0 | 1 | 6 | 0 | 0 |
| 7 | 0 | 0 | 0 | 0 | 0 | 0 | 0 | 14 | 0 | 0 | 0 | 0 |
| 8 | 0 | 0 | 0 | 0 | 0 | 0 | 0 | 0 | 13 | 0 | 0 | 0 |
| 9 | 0 | 0 | 0 | 0 | 0 | 0 | 0 | 0 | 0 | 17 | 1 | 0 |
| 10 | 0 | 0 | 0 | 0 | 0 | 0 | 0 | 0 | 0 | 0 | 14 | 0 |
| 11 | 0 | 0 | 0 | 0 | 0 | 0 | 0 | 0 | 0 | 0 | 0 | 15 |

**Supplementary Table 18** : Confusion matrix for 12-class canine sarcoma classification

## Scenario B

| | Model 1 | | Model 2 | | Model 3 | |
|---|---|---|---|---|---|---|
| | Accuracy = 0.95 ± 0.02 | | Accuracy = 0.99 ± 0.00 | | 0.96 ± 0.00 ± 0.01 | |
| **Index** | **Sensitivity** | **Specificity** | **Sensitivity** | **Specificity** | **Sensitivity** | **Specificity** |
| 0 | 0.954 | 0.993 | 0.989 | 1.000 | 0.976 | 0.996 |
| 1 | 1.000 | 1.000 | 1.000 | 1.000 | 1.000 | 1.000 |
| 2 | 0.990 | 0.998 | 1.000 | 1.000 | 0.971 | 1.000 |
| 3 | 1.000 | 0.998 | 1.000 | 1.000 | 1.000 | 0.997 |
| 4 | 1.000 | 1.000 | 1.000 | 1.000 | 1.000 | 1.000 |
| 5 | 0.955 | 0.996 | 1.000 | 1.000 | 0.982 | 1.000 |
| 6 | 0.951 | 0.970 | 0.987 | 1.000 | 1.000 | 0.984 |
| 7 | 1.000 | 1.000 | 1.000 | 1.000 | 1.000 | 1.000 |
| 8 | 0.882 | 1.000 | 1.000 | 1.000 | 0.900 | 1.000 |
| 9 | 0.793 | 0.993 | 1.000 | 0.997 | 0.900 | 0.997 |
| 10 | 0.894 | 1.000 | 1.000 | 1.000 | 1.000 | 1.000 |
| 11 | 1.000 | 1.000 | 1.000 | 1.000 | 1.000 | 1.000 |

**Supplementary Table 19** : Overall accuracy, sensitivity, and specificity values for 12-class canine sarcoma classification

**Model 1**

| Actual \ Predicted | 0 | 1 | 2 | 3 | 4 | 5 | 6 | 7 | 8 | 9 | 10 | 11 |
|---|---|---|---|---|---|---|---|---|---|---|---|---|
| 0 | 87 | 0 | 0 | 0 | 0 | 0 | 2 | 0 | 0 | 1 | 0 | 0 |
| 1 | 0 | 17 | 0 | 0 | 0 | 0 | 0 | 0 | 0 | 0 | 0 | 0 |
| 2 | 0 | 0 | 92 | 0 | 0 | 1 | 0 | 0 | 0 | 0 | 0 | 0 |
| 3 | 0 | 0 | 0 | 37 | 0 | 1 | 0 | 0 | 0 | 0 | 0 | 0 |
| 4 | 0 | 0 | 0 | 0 | 17 | 0 | 0 | 0 | 0 | 0 | 0 | 0 |
| 5 | 0 | 0 | 0 | 0 | 0 | 86 | 2 | 0 | 0 | 0 | 0 | 0 |
| 6 | 6 | 0 | 1 | 0 | 0 | 2 | 98 | 0 | 0 | 5 | 1 | 0 |
| 7 | 0 | 0 | 0 | 0 | 0 | 0 | 0 | 19 | 0 | 0 | 0 | 0 |
| 8 | 0 | 0 | 0 | 0 | 0 | 0 | 0 | 0 | 15 | 0 | 0 | 0 |
| 9 | 0 | 0 | 0 | 0 | 0 | 0 | 1 | 0 | 2 | 23 | 1 | 0 |
| 10 | 0 | 0 | 0 | 0 | 0 | 0 | 0 | 0 | 0 | 0 | 17 | 0 |
| 11 | 0 | 0 | 0 | 0 | 0 | 0 | 0 | 0 | 0 | 0 | 0 | 19 |

**Model 2**

| Actual \ Predicted | 0 | 1 | 2 | 3 | 4 | 5 | 6 | 7 | 8 | 9 | 10 | 11 |
|---|---|---|---|---|---|---|---|---|---|---|---|---|
| 0 | 93 | 0 | 0 | 0 | 0 | 0 | 0 | 0 | 0 | 0 | 0 | 0 |
| 1 | 0 | 10 | 0 | 0 | 0 | 0 | 0 | 0 | 0 | 0 | 0 | 0 |
| 2 | 0 | 0 | 80 | 0 | 0 | 0 | 0 | 0 | 0 | 0 | 0 | 0 |
| 3 | 0 | 0 | 0 | 26 | 0 | 0 | 0 | 0 | 0 | 0 | 0 | 0 |
| 4 | 0 | 0 | 0 | 0 | 12 | 0 | 0 | 0 | 0 | 0 | 0 | 0 |
| 5 | 0 | 0 | 0 | 0 | 0 | 69 | 0 | 0 | 0 | 0 | 0 | 0 |
| 6 | 1 | 0 | 0 | 0 | 0 | 0 | 78 | 0 | 0 | 0 | 0 | 0 |
| 7 | 0 | 0 | 0 | 0 | 0 | 0 | 0 | 13 | 0 | 0 | 0 | 0 |
| 8 | 0 | 0 | 0 | 0 | 0 | 0 | 0 | 0 | 13 | 0 | 0 | 0 |
| 9 | 0 | 0 | 0 | 0 | 0 | 0 | 1 | 0 | 0 | 20 | 0 | 0 |
| 10 | 0 | 0 | 0 | 0 | 0 | 0 | 0 | 0 | 0 | 0 | 14 | 0 |
| 11 | 0 | 0 | 0 | 0 | 0 | 0 | 0 | 0 | 0 | 0 | 0 | 14 |

**Model 3**

| Actual \ Predicted | 0 | 1 | 2 | 3 | 4 | 5 | 6 | 7 | 8 | 9 | 10 | 11 |
|---|---|---|---|---|---|---|---|---|---|---|---|---|
| 0 | 89 | 0 | 1 | 0 | 0 | 0 | 0 | 0 | 0 | 0 | 0 | 0 |
| 1 | 0 | 12 | 0 | 0 | 0 | 0 | 0 | 0 | 0 | 0 | 0 | 0 |
| 2 | 0 | 0 | 67 | 0 | 0 | 0 | 0 | 0 | 0 | 0 | 0 | 0 |
| 3 | 0 | 0 | 1 | 22 | 0 | 0 | 0 | 0 | 0 | 0 | 0 | 0 |
| 4 | 0 | 0 | 0 | 0 | 11 | 0 | 0 | 0 | 0 | 0 | 0 | 0 |
| 5 | 0 | 0 | 0 | 0 | 0 | 56 | 0 | 0 | 0 | 0 | 0 | 0 |
| 6 | 1 | 0 | 0 | 0 | 0 | 1 | 64 | 0 | 1 | 2 | 0 | 0 |
| 7 | 0 | 0 | 0 | 0 | 0 | 0 | 0 | 11 | 0 | 0 | 0 | 0 |
| 8 | 0 | 0 | 0 | 0 | 0 | 0 | 0 | 0 | 9 | 0 | 0 | 0 |
| 9 | 1 | 0 | 0 | 0 | 0 | 0 | 0 | 0 | 0 | 18 | 0 | 0 |
| 10 | 0 | 0 | 0 | 0 | 0 | 0 | 0 | 0 | 0 | 0 | 12 | 0 |
| 11 | 0 | 0 | 0 | 0 | 0 | 0 | 0 | 0 | 0 | 0 | 0 | 12 |

**Supplementary Table 20** : Confusion matrix for 12-class canine sarcoma classification

## External public datasets results

| Model 2 | | Transfer learning | |
|---|---|---|---|
| Accuracy = 0.78 ± 0.02 | | Accuracy = 0.98 ± 0.00 | |
| **Sensitivity** | **Specificity** | **Sensitivity** | **Specificity** |
| 0.809 | 0.769 | 1.000 | 0.967 |

**Supplementary Table 21** : Overall global accuracy, sensitivity, and specificity values for 2-class human ovary 1 classification

| **Model 2** | | Predicted | |
|---|---|---|---|
| | | 0 | 1 |
| Actual | 0 | 17 | 6 |
| | 1 | 4 | 20 |

| **Transfer learning** | | Predicted | |
|---|---|---|---|
| | | 0 | 1 |
| Actual | 0 | 18 | 0 |
| | 1 | 0 | 25 |

**Supplementary Table 22** : Confusion matrix for 2-class human ovary 1 classification

| Model 2 | | Transfer learning | | cumulative learning | |
|---|---|---|---|---|---|
| Accuracy = 0.80 ± 0.00 | | Accuracy = 0.83 ± 0.02 | | Accuracy = 0.99 ± 0.00 | |
| **Sensitivity** | **Specificity** | **Sensitivity** | **Specificity** | **Sensitivity** | **Specificity** |
| 0.933 | 0.750 | 1.000 | 0.735 | 1.000 | 1.000 |

**Supplementary Table 23** : Overall global accuracy, sensitivity, and specificity values for 2-class human ovary 2 classification

| **Model 2** | | Predicted | |
|---|---|---|---|
| | | 0 | 1 |
| Actual | 0 | 14 | 9 |
| | 1 | 1 | 27 |

| **Transfer learning** | | Predicted | |
|---|---|---|---|
| | | 0 | 1 |
| Actual | 0 | 20 | 9 |
| | 1 | 0 | 25 |

| **cumulative learning** | | Predicted | |
|---|---|---|---|
| | | 0 | 1 |
| Actual | 0 | 20 | 0 |
| | 1 | 0 | 29 |

**Supplementary Table 24** : Confusion matrix for 2-class human ovary 2 classification

## Comparison of our 1D-CNNs against ML approaches

| SVM | RF | LDA |
|---|---|---|
| Kernel : radial<br>C ∈ [−0.01, 0.1, 1, 10]<br>$\gamma$∈ [0.0001, 0.001, 0.01] | max_depth : 2<br>max_features ∈ [0.5 to 0.9]<br>n_estimators ∈ [10 to 100] | # components ∈ [2 to 10]<br>tolerance = $1.e^{-4}$ |

**Supplementary Table 25** : Optimized hyper-parameters for SVM, RF, and LDA algorithms

## Applied to raw datasets

| SVM | | RF | | LDA | |
|---|---|---|---|---|---|
| Accuracy = 0.77 ± 0.02 | | Accuracy = 0.96 ± 0.01 | | Accuracy = 0.71 ± 0.02 | |
| **Sensitivity** | **Specificity** | **Sensitivity** | **Specificity** | **Sensitivity** | **Specificity** |
| 0.055 | 0.920 | 0.836 | 1.000 | 0.961 | 0.637 |

**Supplementary Table 26** : Overall accuracy, sensitivity, and specificity values for 2-class canine sarcoma classification

**SVM**

| | | Predicted 0 | Predicted 1 |
|---|---|---|---|
| Actual | 0 | 5 | 25 |
| | 1 | 90 | 327 |

**RF**

| | | Predicted 0 | Predicted 1 |
|---|---|---|---|
| Actual | 0 | 80 | 0 |
| | 1 | 18 | 359 |

**LDA**

| | | Predicted 0 | Predicted 1 |
|---|---|---|---|
| Actual | 0 | 91 | 120 |
| | 1 | 3 | 213 |

**Supplementary Table 27** : Confusion matrix for 2-class canine sarcoma classification

| | SVM | | RF | | LDA | |
|---|---|---|---|---|---|---|
| | Accuracy = 0.61 ± 0.00 | | Accuracy =0.65 ± 0.04 | | Accuracy = 0.41 ± 0.01 | |
| **Index** | **Sensitivity** | **Specificity** | **Sensitivity** | **Specificity** | **Sensitivity** | **Specificity** |
| 0 | 1.000 | 0.875 | 1.000 | 0.875 | 0.476 | 0.956 |
| 1 | 0.333 | 1.000 | 0.333 | 1.000 | 0.750 | 0.968 |
| 2 | 0.738 | 0.817 | 0.738 | 0.817 | 0.354 | 0.865 |
| 3 | 0.083 | 1.000 | 0.083 | 1.000 | 0.133 | 0.982 |
| 4 | 0.000 | 1.000 | 0.000 | 1.000 | 0.800 | 0.952 |
| 5 | 0.707 | 0.890 | 0.707 | 0.890 | 0.190 | 0.983 |
| 6 | 0.602 | 0.976 | 0.602 | 0.976 | 0.187 | 0.958 |
| 7 | 0.818 | 1.000 | 0.818 | 1.000 | 0.421 | 0.934 |
| 8 | 0.818 | 1.000 | 0.428 | 1.000 | 0.764 | 0.752 |
| 9 | 0.296 | 0.997 | 0.296 | 0.997 | 0.291 | 0.980 |
| 10 | 0.600 | 1.000 | 0.600 | 1.000 | 0.375 | 0.983 |
| 11 | 0.545 | 1.000 | 0.545 | 1.000 | 0.846 | 0.992 |

**Supplementary Table 28** : Overall accuracy, sensitivity, and specificity values for 12-class canine sarcoma classification

**SVM**

| Actual \ Predicted | 0 | 1 | 2 | 3 | 4 | 5 | 6 | 7 | 8 | 9 | 10 | 11 |
|---|---|---|---|---|---|---|---|---|---|---|---|---|
| 0 | 97 | 3 | 2 | 0 | 0 | 0 | 15 | 1 | 6 | 17 | 1 | 1 |
| 1 | 0 | 5 | 0 | 0 | 0 | 0 | 0 | 0 | 0 | 0 | 0 | 0 |
| 2 | 0 | 7 | 65 | 18 | 13 | 18 | 6 | 1 | 0 | 0 | 2 | 4 |
| 3 | 0 | 0 | 0 | 3 | 0 | 0 | 0 | 0 | 0 | 0 | 0 | 0 |
| 4 | 0 | 0 | 0 | 0 | 0 | 0 | 0 | 0 | 0 | 0 | 0 | 0 |
| 5 | 0 | 0 | 18 | 15 | 2 | 46 | 9 | 0 | 0 | 0 | 0 | 0 |
| 6 | 0 | 0 | 3 | 0 | 0 | 1 | 47 | 0 | 2 | 2 | 1 | 0 |
| 7 | 0 | 0 | 0 | 0 | 0 | 0 | 0 | 9 | 0 | 0 | 0 | 0 |
| 8 | 0 | 0 | 0 | 0 | 0 | 0 | 0 | 0 | 6 | 0 | 0 | 0 |
| 9 | 0 | 0 | 0 | 0 | 0 | 0 | 1 | 0 | 0 | 8 | 0 | 0 |
| 10 | 0 | 0 | 0 | 0 | 0 | 0 | 0 | 0 | 0 | 0 | 6 | 0 |
| 11 | 0 | 0 | 0 | 0 | 0 | 0 | 0 | 0 | 0 | 0 | 0 | 6 |

**RF**

| Actual \ Predicted | 0 | 1 | 2 | 3 | 4 | 5 | 6 | 7 | 8 | 9 | 10 | 11 |
|---|---|---|---|---|---|---|---|---|---|---|---|---|
| 0 | 97 | 3 | 2 | 0 | 0 | 0 | 15 | 1 | 6 | 17 | 1 | 1 |
| 1 | 0 | 5 | 0 | 0 | 0 | 0 | 0 | 0 | 0 | 0 | 0 | 0 |
| 2 | 0 | 7 | 65 | 18 | 13 | 18 | 6 | 1 | 0 | 0 | 2 | 4 |
| 3 | 0 | 0 | 0 | 3 | 0 | 0 | 0 | 0 | 0 | 0 | 0 | 0 |
| 4 | 0 | 0 | 0 | 0 | 0 | 0 | 0 | 0 | 0 | 0 | 0 | 0 |
| 5 | 0 | 0 | 18 | 15 | 2 | 46 | 9 | 0 | 0 | 0 | 0 | 0 |
| 6 | 0 | 0 | 3 | 0 | 0 | 1 | 47 | 0 | 2 | 2 | 1 | 0 |
| 7 | 0 | 0 | 0 | 0 | 0 | 0 | 0 | 9 | 0 | 0 | 0 | 0 |
| 8 | 0 | 0 | 0 | 0 | 0 | 0 | 0 | 0 | 6 | 0 | 0 | 0 |
| 9 | 0 | 0 | 0 | 0 | 0 | 0 | 1 | 0 | 0 | 8 | 0 | 0 |
| 10 | 0 | 0 | 0 | 0 | 0 | 0 | 0 | 0 | 0 | 0 | 6 | 0 |
| 11 | 0 | 0 | 0 | 0 | 0 | 0 | 0 | 0 | 0 | 0 | 0 | 6 |

**LDA**

| Actual \ Predicted | 0 | 1 | 2 | 3 | 4 | 5 | 6 | 7 | 8 | 9 | 10 | 11 |
|---|---|---|---|---|---|---|---|---|---|---|---|---|
| 0 | 41 | 0 | 4 | 0 | 0 | 5 | 5 | 0 | 0 | 0 | 1 | 0 |
| 1 | 1 | 12 | 1 | 2 | 1 | 5 | 3 | 0 | 0 | 0 | 0 | 0 |
| 2 | 2 | 1 | 28 | 6 | 0 | 17 | 16 | 4 | 1 | 0 | 0 | 0 |
| 3 | 0 | 0 | 2 | 4 | 0 | 1 | 4 | 0 | 0 | 0 | 0 | 0 |
| 4 | 0 | 0 | 5 | 9 | 8 | 5 | 0 | 1 | 0 | 0 | 0 | 0 |
| 5 | 0 | 0 | 5 | 0 | 0 | 12 | 1 | 0 | 0 | 0 | 0 | 0 |
| 6 | 5 | 0 | 3 | 2 | 0 | 0 | 12 | 0 | 0 | 5 | 0 | 0 |
| 7 | 0 | 0 | 9 | 3 | 0 | 10 | 5 | 8 | 0 | 0 | 0 | 0 |
| 8 | 33 | 3 | 16 | 4 | 1 | 8 | 16 | 6 | 13 | 12 | 3 | 0 |
| 9 | 1 | 0 | 1 | 0 | 0 | 0 | 0 | 0 | 3 | 7 | 1 | 2 |
| 10 | 0 | 0 | 5 | 0 | 0 | 0 | 2 | 0 | 0 | 0 | 3 | 0 |
| 11 | 3 | 0 | 0 | 0 | 0 | 0 | 0 | 0 | 0 | 0 | 0 | 11 |

**Supplementary Table 29** : Confusion matrix for 12-class canine sarcoma classification

| | SVM | | RF | | LDA | |
|---|---|---|---|---|---|---|
| | Accuracy = 0.45 ± 0.03 | | Accuracy = 0.77 ± 0.03 | | Accuracy = 0.90 ± 0.01 | |
| **Index** | **Sensitivity** | **Specificity** | **Sensitivity** | **Specificity** | **Sensitivity** | **Specificity** |
| 0 | 1.000 | 0.000 | 0.900 | 0.705 | 1.000 | 0.818 |
| 1 | 0.000 | 1.000 | 0.777 | 0.944 | 0.777 | 1.000 |
| 2 | 0.000 | 1.000 | 0.625 | 1.000 | 1.000 | 1.000 |

**Supplementary Table 30** : Overall accuracy, sensitivity, and specificity values for 3-class microorganisms classification

**SVM**

| | | Predicted | | |
|---|---|---|---|---|
| | | 0 | 1 | 2 |
| Actual | 0 | 11 | 9 | 4 |
| | 1 | 0 | 0 | 0 |
| | 2 | 0 | 0 | 0 |

**RF**

| | | Predicted | | |
|---|---|---|---|---|
| | | 0 | 1 | 2 |
| Actual | 0 | 9 | 2 | 3 |
| | 1 | 1 | 7 | 0 |
| | 2 | 0 | 0 | 5 |

**LDA**

| | | Predicted | | |
|---|---|---|---|---|
| | | 0 | 1 | 2 |
| Actual | 0 | 10 | 2 | 0 |
| | 1 | 0 | 7 | 0 |
| | 2 | 0 | 0 | 5 |

**Supplementary Table 31** : Confusion matrix for 3-class microorganisms classification

| | SVM | | RF | | LDA | |
|---|---|---|---|---|---|---|
| | Accuracy = 0.54 ± 0.35 | | Accuracy = 0.86 ± 0.01 | | Accuracy = 0.67 ± 0.13 | |
| **Index** | **Sensitivity** | **Specificity** | **Sensitivity** | **Specificity** | **Sensitivity** | **Specificity** |
| 0 | 1.000 | 0.588 | 1.000 | 0.933 | 0.631 | 1.000 |
| 1 | 0.000 | 1.000 | 1.000 | 1.000 | 0.000 | 1.000 |
| 2 | 0.667 | 1.000 | 0.666 | 0.937 | 0.755 | 0.615 |
| 3 | 0.000 | 1.000 | 0.666 | 1.000 | 1.000 | 1.000 |
| 4 | 1.000 | 0.842 | 1.000 | 1.000 | 0.500 | 0.840 |

**Supplementary Table 32** : Overall accuracy, sensitivity, and specificity values for 5-class microorganisms classification

**SVM**

| | | Predicted | | | | |
|---|---|---|---|---|---|---|
| | | 0 | 1 | 2 | 3 | 4 |
| Actual | 0 | 4 | 7 | 4 | 2 | 6 |
| | 1 | 0 | 0 | 4 | 0 | 0 |
| | 2 | 0 | 0 | 4 | 0 | 0 |
| | 3 | 0 | 0 | 4 | 0 | 0 |
| | 4 | 0 | 0 | 4 | 0 | 0 |

**RF**

| | | Predicted | | | | |
|---|---|---|---|---|---|---|
| | | 0 | 1 | 2 | 3 | 4 |
| Actual | 0 | 3 | 1 | 1 | 0 | 1 |
| | 1 | 0 | 2 | 0 | 0 | 0 |
| | 2 | 0 | 0 | 4 | 0 | 0 |
| | 3 | 0 | 0 | 0 | 6 | 0 |
| | 4 | 0 | 0 | 0 | 0 | 2 |

**LDA**

| | | Predicted | | | | |
|---|---|---|---|---|---|---|
| | | 0 | 1 | 2 | 3 | 4 |
| Actual | 0 | 7 | 0 | 0 | 0 | 0 |
| | 1 | 0 | 5 | 0 | 0 | 0 |
| | 2 | 1 | 2 | 4 | 0 | 0 |
| | 3 | 0 | 0 | 1 | 3 | 0 |
| | 4 | 0 | 0 | 0 | 0 | 4 |

**Supplementary Table 33** : Confusion matrix for 5-class microorganisms classification

| SVM | | RF | | LDA | |
|---|---|---|---|---|---|
| Accuracy = 0.53 ± 0.04 | | Accuracy = 0.84 ± 0.05 | | Accuracy = 0.65 ± 0.04 | |
| **Sensitivity** | **Specificity** | **Sensitivity** | **Specificity** | **Sensitivity** | **Specificity** |
| 0.210 | 0.990 | 0.760 | 0.952 | 0.666 | 0.636 |

**Supplementary Table 34** : Overall accuracy, sensitivity, and specificity values for 2-class human ovary 1 classification

**SVM**

| | | Predicted | |
|---|---|---|---|
| | | 0 | 1 |
| Actual | 0 | 3 | 1 |
| | 1 | 21 | 24 |

**RF**

| | | Predicted | |
|---|---|---|---|
| | | 0 | 1 |
| Actual | 0 | 19 | 1 |
| | 1 | 6 | 20 |

**LDA**

| | | Predicted | |
|---|---|---|---|
| | | 0 | 1 |
| Actual | 0 | 18 | 8 |
| | 1 | 9 | 14 |

**Supplementary Table 35** : Confusion matrix for 2-class human ovary 1 classification

| SVM | | RF | | LDA | |
|---|---|---|---|---|---|
| Accuracy = 0.60 ± 0.06 | | Accuracy = 0.81 ± 0.01 | | Accuracy = 0.71 ± 0.03 | |
| **Sensitivity** | **Specificity** | **Sensitivity** | **Specificity** | **Sensitivity** | **Specificity** |
| 0.213 | 0.910 | 0.650 | 0.933 | 0.232 | 0.968 |

**Supplementary Table 36** : Overall accuracy, sensitivity, and specificity values for 2-class human ovary 2 classification

**SVM**

| | | Predicted | |
|---|---|---|---|
| | | 0 | 1 |
| Actual | 0 | 2 | 3 |
| | 1 | 19 | 29 |

**RF**

| | | Predicted | |
|---|---|---|---|
| | | 0 | 1 |
| Actual | 0 | 13 | 2 |
| | 1 | 7 | 28 |

**LDA**

| | | Predicted | |
|---|---|---|---|
| | | 0 | 1 |
| Actual | 0 | 4 | 2 |
| | 1 | 10 | 31 |

**Supplementary Table 37** : Confusion matrix for 2-class human ovary 2 classification

## Applied to preprocessed datasets

| SVM | | RF | | LDA | |
|---|---|---|---|---|---|
| Accuracy = 0.76 ± 0.16 | | Accuracy = 0.96 ± 0.01 | | Accuracy = 0.93 ± 0.02 | |
| **Sensitivity** | **Specificity** | **Sensitivity** | **Specificity** | **Sensitivity** | **Specificity** |
| 0.465 | 1.000 | 0.850 | 0.994 | 0.970 | 0.930 |

**Supplementary Table 38** : Overall accuracy, sensitivity, and specificity values for 2-class canine sarcoma classification

**SVM**

| | | Predicted 0 | Predicted 1 |
|---|---|---|---|
| Actual | 0 | 54 | 0 |
| Actual | 1 | 62 | 343 |

**RF**

| | | Predicted 0 | Predicted 1 |
|---|---|---|---|
| Actual | 0 | 74 | 2 |
| Actual | 1 | 13 | 341 |

**LDA**

| | | Predicted 0 | Predicted 1 |
|---|---|---|---|
| Actual | 0 | 97 | 25 |
| Actual | 1 | 4 | 329 |

**Supplementary Table 39** : Confusion matrix for 2-class canine sarcoma classification

| | SVM | | RF | | LDA | |
|---|---|---|---|---|---|---|
| | Accuracy = 0.52 ± 0.19 | | Accuracy = 0.65 ± 0.01 | | Accuracy = 0.60 ± 0.04 | |
| **Index** | **Sensitivity** | **Specificity** | **Sensitivity** | **Specificity** | **Sensitivity** | **Specificity** |
| 0 | 1.000 | 0.766 | 0.963 | 0.966 | 1.000 | 0.809 |
| 1 | 0.800 | 0.988 | 0.375 | 1.000 | 0.909 | 1.000 |
| 2 | 0.378 | 0.942 | 0.725 | 1.000 | 0.764 | 0.932 |
| 3 | 0.423 | 0.958 | 0.086 | 1.000 | 0.833 | 1.000 |
| 4 | 0.000 | 1.000 | 0.000 | 1.000 | 0.909 | 1.000 |
| 5 | 0.507 | 0.913 | 0.645 | 1.000 | 0.485 | 0.994 |
| 6 | 0.454 | 0.949 | 0.675 | 1.000 | 0.434 | 0.997 |
| 7 | 0.000 | 1.000 | 0.615 | 1.000 | 0.000 | 1.000 |
| 8 | 0.083 | 1.000 | 0.583 | 0.835 | 0.000 | 1.000 |
| 9 | 0.565 | 0.933 | 0.363 | 1.000 | 0.000 | 1.000 |
| 10 | 0.000 | 1.000 | 0.600 | 1.000 | 0.800 | 0.822 |
| 11 | 0.384 | 0.993 | 0.461 | 0.920 | 0.000 | 1.000 |

**Supplementary Table 40** : Overall accuracy, sensitivity, and specificity values for 12-class canine sarcoma classification

**SVM** (Predicted)

| Actual | 0 | 1 | 2 | 3 | 4 | 5 | 6 | 7 | 8 | 9 | 10 | 11 |
|---|---|---|---|---|---|---|---|---|---|---|---|---|
| 0 | 98 | 3 | 21 | 7 | 0 | 11 | 15 | 12 | 3 | 8 | 2 | 3 |
| 1 | 0 | 12 | 1 | 0 | 0 | 3 | 1 | 0 | 0 | 0 | 0 | 0 |
| 2 | 0 | 0 | 31 | 0 | 0 | 3 | 15 | 0 | 1 | 0 | 2 | 1 |
| 3 | 0 | 0 | 7 | 11 | 1 | 8 | 2 | 0 | 0 | 0 | 0 | 0 |
| 4 | 0 | 0 | 0 | 0 | 0 | 0 | 0 | 0 | 0 | 0 | 0 | 0 |
| 5 | 0 | 0 | 14 | 6 | 7 | 36 | 3 | 0 | 0 | 0 | 0 | 4 |
| 6 | 0 | 0 | 4 | 2 | 1 | 10 | 40 | 0 | 0 | 2 | 0 | 0 |
| 7 | 0 | 0 | 0 | 0 | 0 | 0 | 0 | 0 | 0 | 0 | 0 | 0 |
| 8 | 0 | 0 | 0 | 0 | 0 | 0 | 0 | 0 | 1 | 0 | 0 | 0 |
| 9 | 0 | 0 | 4 | 0 | 0 | 0 | 12 | 1 | 7 | 13 | 5 | 0 |
| 10 | 0 | 0 | 0 | 0 | 0 | 0 | 0 | 0 | 0 | 0 | 0 | 0 |
| 11 | 0 | 0 | 0 | 0 | 0 | 0 | 0 | 0 | 0 | 0 | 3 | 5 |

**RF** (Predicted)

| Actual | 0 | 1 | 2 | 3 | 4 | 5 | 6 | 7 | 8 | 9 | 10 | 11 |
|---|---|---|---|---|---|---|---|---|---|---|---|---|
| 0 | 79 | 6 | 4 | 1 | 1 | 2 | 13 | 2 | 4 | 12 | 5 | 6 |
| 1 | 0 | 6 | 0 | 0 | 0 | 0 | 0 | 0 | 0 | 0 | 0 | 0 |
| 2 | 1 | 4 | 58 | 16 | 14 | 17 | 2 | 2 | 0 | 0 | 0 | 1 |
| 3 | 0 | 0 | 0 | 2 | 0 | 0 | 0 | 0 | 0 | 0 | 0 | 0 |
| 4 | 0 | 0 | 0 | 0 | 0 | 0 | 0 | 0 | 0 | 0 | 0 | 0 |
| 5 | 0 | 0 | 16 | 4 | 0 | 40 | 9 | 0 | 0 | 0 | 0 | 0 |
| 6 | 2 | 0 | 2 | 0 | 0 | 3 | 50 | 1 | 1 | 2 | 1 | 0 |
| 7 | 0 | 0 | 0 | 0 | 0 | 0 | 0 | 8 | 0 | 0 | 0 | 0 |
| 8 | 0 | 0 | 0 | 0 | 0 | 0 | 0 | 0 | 7 | 0 | 0 | 0 |
| 9 | 0 | 0 | 0 | 0 | 0 | 0 | 0 | 0 | 0 | 8 | 0 | 0 |
| 10 | 0 | 0 | 0 | 0 | 0 | 0 | 0 | 0 | 0 | 0 | 9 | 0 |
| 11 | 0 | 0 | 0 | 0 | 0 | 0 | 0 | 0 | 0 | 0 | 0 | 6 |

**LDA** (Predicted)

| Actual | 0 | 1 | 2 | 3 | 4 | 5 | 6 | 7 | 8 | 9 | 10 | 11 |
|---|---|---|---|---|---|---|---|---|---|---|---|---|
| 0 | 96 | 0 | 0 | 0 | 0 | 0 | 29 | 12 | 11 | 11 | 2 | 0 |
| 1 | 0 | 10 | 0 | 0 | 0 | 0 | 0 | 0 | 0 | 0 | 0 | 0 |
| 2 | 0 | 1 | 52 | 4 | 1 | 18 | 1 | 0 | 0 | 0 | 0 | 0 |
| 3 | 0 | 0 | 0 | 25 | 0 | 0 | 0 | 0 | 0 | 0 | 0 | 0 |
| 4 | 0 | 0 | 0 | 0 | 10 | 0 | 0 | 0 | 0 | 0 | 0 | 0 |
| 5 | 0 | 0 | 1 | 0 | 0 | 34 | 1 | 0 | 0 | 0 | 0 | 0 |
| 6 | 0 | 0 | 0 | 0 | 0 | 1 | 33 | 0 | 0 | 0 | 0 | 0 |
| 7 | 0 | 0 | 0 | 0 | 0 | 0 | 0 | 0 | 0 | 0 | 0 | 0 |
| 8 | 0 | 0 | 0 | 0 | 0 | 0 | 0 | 0 | 0 | 0 | 0 | 0 |
| 9 | 0 | 0 | 0 | 0 | 0 | 0 | 0 | 0 | 0 | 0 | 0 | 0 |
| 10 | 0 | 0 | 15 | 1 | 0 | 17 | 12 | 0 | 3 | 11 | 8 | 17 |
| 11 | 0 | 0 | 0 | 0 | 0 | 0 | 0 | 0 | 0 | 0 | 0 | 0 |

**Supplementary Table 41** : Confusion matrix for 12-class canine sarcoma classification

| | SVM | | RF | | LDA | |
|---|---|---|---|---|---|---|
| | Accuracy = 0.87 ± 0.04 | | Accuracy = 0.95 ± 0.02 | | Accuracy = 0.88 ± 0.02 | |
| **Index** | **Sensitivity** | **Specificity** | **Sensitivity** | **Specificity** | **Sensitivity** | **Specificity** |
| 0 | 0.964 | 0.807 | 0.922 | 0.914 | 0.777 | 1.000 |
| 1 | 0.720 | 0.975 | 0.853 | 0.955 | 1.000 | 0.919 |
| 2 | 0.930 | 1.000 | 1.000 | 0.993 | 1.000 | 0.931 |

**Supplementary Table 42** : Overall accuracy, sensitivity, and specificity values for 3-class microorganisms classification

**SVM** (Predicted)

| Actual | 0 | 1 | 2 |
|---|---|---|---|
| 0 | 9 | 2 | 0 |
| 1 | 0 | 5 | 0 |
| 2 | 0 | 0 | 4 |

**RF** (Predicted)

| Actual | 0 | 1 | 2 |
|---|---|---|---|
| 0 | 11 | 1 | 0 |
| 1 | 0 | 6 | 0 |
| 2 | 0 | 0 | 4 |

**LDA** (Predicted)

| Actual | 0 | 1 | 2 |
|---|---|---|---|
| 0 | 7 | 0 | 0 |
| 1 | 1 | 5 | 0 |
| 2 | 1 | 0 | 4 |

**Supplementary Table 43** : Confusion matrix for 3-class microorganisms classification

| | SVM | | RF | | LDA | |
|---|---|---|---|---|---|---|
| | Accuracy = 0.19 ± 0.09 | | Accuracy = 0.87 ± 0.02 | | Accuracy = 0.85 ± 0.03 | |
| **Index** | **Sensitivity** | **Specificity** | **Sensitivity** | **Specificity** | **Sensitivity** | **Specificity** |
| 0 | 1.000 | 0.000 | 1.000 | 0.823 | 0.875 | 1.000 |
| 1 | 0.000 | 1.000 | 0.666 | 1.000 | 0.714 | 1.000 |
| 2 | 0.000 | 1.000 | 0.800 | 1.000 | 0.800 | 0.863 |
| 3 | 0.000 | 1.000 | 1.000 | 1.000 | 1.000 | 0.958 |
| 4 | 0.000 | 1.000 | 0.666 | 1.000 | 1.000 | 1.000 |

**Supplementary Table 44** : Overall accuracy, sensitivity, and specificity values for 5-class microorganisms classification

| **SVM** | | Predicted | | | | |
|---|---|---|---|---|---|---|
| | | 0 | 1 | 2 | 3 | 4 |
| Actual | 0 | 5 | 3 | 2 | 4 | 0 |
| | 1 | 0 | 0 | 0 | 0 | 0 |
| | 2 | 0 | 0 | 4 | 0 | 0 |
| | 3 | 0 | 0 | 0 | 0 | 0 |
| | 4 | 0 | 3 | 0 | 0 | 4 |

| **RF** | | Predicted | | | | |
|---|---|---|---|---|---|---|
| | | 0 | 1 | 2 | 3 | 4 |
| Actual | 0 | 4 | 0 | 1 | 0 | 0 |
| | 1 | 0 | 5 | 0 | 0 | 0 |
| | 2 | 0 | 0 | 3 | 1 | 0 |
| | 3 | 0 | 0 | 0 | 3 | 0 |
| | 4 | 0 | 0 | 0 | 0 | 4 |

| **LDA** | | Predicted | | | | |
|---|---|---|---|---|---|---|
| | | 0 | 1 | 2 | 3 | 4 |
| Actual | 0 | 5 | 0 | 0 | 0 | 0 |
| | 1 | 0 | 0 | 0 | 0 | 0 |
| | 2 | 2 | 5 | 3 | 0 | 3 |
| | 3 | 0 | 0 | 0 | 5 | 0 |
| | 4 | 1 | 3 | 1 | 0 | 2 |

**Supplementary Table 45** : Confusion matrix for 5-class microorganisms classification

| SVM | | RF | | LDA | |
|---|---|---|---|---|---|
| Accuracy = 0.66 ± 0.24 | | Accuracy = 0.91 ± 0.02 | | Accuracy = 0.93 ± 0.02 | |
| **Sensitivity** | **Specificity** | **Sensitivity** | **Specificity** | **Sensitivity** | **Specificity** |
| 0.000 | 1.000 | 1.000 | 0.818 | 0.934 | 0.956 |

**Supplementary Table 46** : Overall accuracy, sensitivity, and specificity values for 2-class human ovary 1 classification

| **SVM** | | Predicted | |
|---|---|---|---|
| | | 0 | 1 |
| Actual | 0 | 0 | 0 |
| | 1 | 18 | 35 |

| **RF** | | Predicted | |
|---|---|---|---|
| | | 0 | 1 |
| Actual | 0 | 19 | 4 |
| | 1 | 0 | 18 |

| **LDA** | | Predicted | |
|---|---|---|---|
| | | 0 | 1 |
| Actual | 0 | 16 | 1 |
| | 1 | 2 | 28 |

**Supplementary Table 47** : Confusion matrix for 2-class human ovary 1 classification

| SVM | | RF | | LDA | |
|---|---|---|---|---|---|
| Accuracy = 0.60 ± 0.05 | | Accuracy = 0.88 ± 0.03 | | Accuracy = 0.96 ± 0.00 | |
| **Sensitivity** | **Specificity** | **Sensitivity** | **Specificity** | **Sensitivity** | **Specificity** |
| 0.000 | 1.000 | 0.761 | 0.968 | 0.990 | 0.963 |

**Supplementary Table 48** : Overall accuracy, sensitivity, and specificity values for 2-class human ovary 2 classification

| **SVM** | | Predicted | |
|---|---|---|---|
| | | 0 | 1 |
| Actual | 0 | 0 | 0 |
| | 1 | 21 | 32 |

| **RF** | | Predicted | |
|---|---|---|---|
| | | 0 | 1 |
| Actual | 0 | 16 | 1 |
| | 1 | 5 | 31 |

| **LDA** | | Predicted | |
|---|---|---|---|
| | | 0 | 1 |
| Actual | 0 | 19 | 1 |
| | 1 | 1 | 26 |

**Supplementary Table 49** : Confusion matrix for 2-class human ovary 2 classification

## CNN architectures performances with 1D-MS data binned to 1

We follow the same training and evaluation protocol as described previously. The results of the binning at 1 of the SpiderMass datasets : Canine sarcoma (1500 features) and Microorganisms (1900 features) are not considered in our study as this bin size is too aggressive and reduce considerably the spectra information but the results of accuracies are nevertheless shown below.

| Datasets | # classes | variant_Lecun | variant_LeNet | variant_VGG9 |
|---|---|---|---|---|
| Canine sarcoma | 2 | **0.98 ± 0.01** | 0.91 ± 0.05 | 0.95 ± 0.02 |
| | 12 | 0.66 ± 0.02 | 0.68 ± 0.08 | **0.72 ± 0.02** |
| Microorganisms | 3 | **0.90 ± 0.01** | 0.69 ± 0.02 | 0.52 ± 0.02 |
| | 5 | **0.75 ± 0.02** | 0.73 ± 0.09 | 0.31 ± 0.06 |

**Supplementary Table 50** : Overall accuracies of SpiderMass spectra classification using three CNN architectures. The best result for each task (accuracy ± standard variation over 10 independent iterations) is indicated in boldface.

| Datasets | # classes | variant_Lecun | variant_LeNet | variant_VGG9 |
|---|---|---|---|---|
| Canine sarcoma | 12 | 0.81 ± 0.00 (22%) | **0.88 ± 0.03** (29%) | 0.87 ± 0.05 (20%) |
| Microorganisms | 3 | **0.99 ± 0.00** (10%) | 0.95 ± 0.01 (37%) | 0.61 ± 0.01 (17%) |
| | 5 | **0.99 ± 0.00** (32%) | **0.99 ± 0.00** (35%) | 0.81 ± 0.02 (161%) |

**Supplementary Table 51** : Overall accuracies of classification of SpiderMass spectra using three CNN architectures after transfer learning. The best result for each task (accuracy ± standard variation over 10 independent iterations) is indicated in boldface. The improvement in performance from scratch is expressed as a percentage.

| Protocol | variant_Lecun | variant_LeNet | variant_VGG9 |
|---|---|---|---|
| Scenario A | 0.86 ± 0.02 (30%* 06%**) | **0.95 ± 0.03** (39%* 07%**) | 0.93 ± 0.02 (29%* 06%**) |
| Scenario B | 0.90 ± 0.01 (36%* 11%** 04%***) | 0.97 ± 0.00 (42%* 10%** 02%***) | **0.98 ± 0.00** (36%* 12%** 05%***) |

**Supplementary Table 52** : Overall accuracies of canine sarcoma classification by the three CNN architectures. The best result for each task (accuracy ± standard variation over 10 independent iterations) is indicated in boldface. The improvement is expressed as a percentage relative to learning from scratch*, to transfer learning**, and to Scenario A***.

## ML approaches performances with 1D-MS data binned to 1

| | | Applied to raw datasets | | | Applied to preprocessed datasets | | |
|---|---|---|---|---|---|---|---|
| Datasets | # classes | SVM | RF | LDA | SVM | RF | LDA |
| Canine sarcoma | 2 | 0.86 ± 0.03 | **0.98 ± 0.01** | 0.62 ± 0.02 | 0.91 ± 0.02 | **0.95 ± 0.10** | 0.91 ± 0.02 |
| | 12 | 0.51 ± 0.07 | **0.69 ± 0.03** | 0.40 ± 0.05 | 0.53 ± 0.22 | **0.75 ± 0.21** | 0.72 ± 0.02 |
| Microorganisms | 3 | 0.39 ± 0.09 | **0.87 ± 0.04** | 0.84 ± 0.07 | 0.88 ± 0.02 | **0.91 ± 0.00** | 0.88 ± 0.01 |
| | 5 | 0.67 ± 0.11 | **0.86 ± 0.03** | 0.82 ± 0.03 | 0.88 ± 0.01 | **0.99 ± 0.01** | 0.85 ± 0.03 |

**Supplementary Table 53** : Overall accuracies of raw and preprocessed clinical spectra classifications by SVM, RF, and LDA. The best result for each task (accuracy ± standard variation over 10 independent iterations) is indicated in boldface.

## Canine spectra correlations

To confirm that our way of splitting the spectra is not favouring a too positive classification outcome, we compute classification accuracies for the 4 canine sarcoma classes containing at least 3 biopsies: Healthy (8 biopsies), Fibrosarcoma (6 biopsies), Osteosarcoma (5 biopsies), Undifferentiated pleomorphic sarcoma (UPS, 5 biopsies) to assess the effect of spectra splitting on the performance of CNN models. We follow the same protocol for training CNNs models.

| | **Split 1** | | | |
|---|---|---|---|---|
| | Model 1 : Accuracy = 0.91 ± 0.04 | | | |
| | **Healthy** | **Fibrosarcoma** | **Osteosarcoma** | **UPS** |
| **Healthy** | 99 | 0 | 0 | 0 |
| **Fibrosarcoma** | 0 | 68 | 8 | 2 |
| **Osteosarcoma** | 0 | 2 | 46 | 2 |
| **UPS** | 4 | 1 | 2 | 62 |
| | Model 2 : Accuracy = 0.88 ± 0.03 | | | |
| **Healthy** | 98 | 0 | 0 | 2 |
| **Fibrosarcoma** | 1 | 60 | 7 | 6 |
| **Osteosarcoma** | 1 | 4 | 59 | 10 |
| **UPS** | 4 | 0 | 0 | 71 |
| | Model 3 : Accuracy = 0.91 ± 0.02 | | | |
| **Healthy** | 110 | 0 | 0 | 4 |
| **Fibrosarcoma** | 0 | 69 | 11 | 1 |
| **Osteosarcoma** | 0 | 5 | 54 | 2 |
| **UPS** | 0 | 3 | 1 | 59 |

| | **Split 2** | | | |
|---|---|---|---|---|
| | Model 1 : Accuracy = 0.93 ± 0.02 | | | |
| | **Healthy** | **Fibrosarcoma** | **Osteosarcoma** | **UPS** |
| **Healthy** | 90 | 0 | 1 | 0 |
| **Fibrosarcoma** | 0 | 73 | 4 | 0 |
| **Osteosarcoma** | 0 | 4 | 65 | 1 |
| **UPS** | 3 | 4 | 4 | 79 |
| | Model 2 : Accuracy = 0.86 ± 0.02 | | | |
| **Healthy** | 74 | 0 | 0 | 0 |
| **Fibrosarcoma** | 0 | 68 | 2 | 1 |
| **Osteosarcoma** | 10 | 13 | 74 | 13 |
| **UPS** | 10 | 2 | 1 | 64 |
| | Model 3 : Accuracy = 0.90 ± 0.01 | | | |
| **Healthy** | 99 | 0 | 3 | 8 |
| **Fibrosarcoma** | 0 | 79 | 1 | 3 |
| **Osteosarcoma** | 0 | 7 | 57 | 5 |
| **UPS** | 3 | 2 | 3 | 63 |

**Supplementary Table 54** : Overall accuracies and confusion matrix. Split 1 : Spectra from the same biopsy are present in either the training set, the validation set or the testing set. Split 2 : Spectra are splitted randomly at each iteration without taking into account their biopsy

As shown in Table 54, classification results are very close and the split used in the paper (split 2) is not favouring a too positive classification outcome.

## 1D-MS Data augmentation

To increase the robustness of the training and compensate for the limited number of SpiderMass spectra, the training set size is increased using the following data augmentation procedure: (1) Random noise proportional to spectrum acquisition order is added. (2) For misalignment, the shift scale is first assessed using a cubic warping function; spectra are then augmented by shifting each one along the m/z dimension using a third-order polynomial randomly between -0.2 and 0.2 Da. (3) Peak intensity values are increased by a random value ranging from 0 to 2 to produce intensity variation and peak absence/presence.

## Computing environment

The proposed 1D-CNNs are computed with Keras library[32] (version 2.2.4) on two NVIDIA P100 Pascal GPUs of 12 GB HBM2 memory (Graham and Cedar supercomputer from Compute Canada at Waterloo university). Models are executed in a Linux environment using the Python language (Python version 3.6.8). Classification models with conventional ML algorithms (SVM, RF, and LDA) are implemented using the Scikit-learn library[33] (version 0.19.1) and are computed on a local CPU server (128 cpus and 1 T. of ram). The computational load and memory requirements for transfer and cumulative learning are low. Indeed, training time on the rat brain dataset is about 25 minutes for variant_Lecun, 50 minutes for variant_LeNet, and 3 hours for variant_VGG. Fine-tuning on clinical datasets is completed in about 20 minute since the weighting coefficients are already determined, and all three architectures processed each test spectrum in less than one millisecond.

# References


1. Friedland, G. *et al.* A practical approach to sizing neural networks. *arXiv preprint arXiv:1810.02328* (2018).
2. Neyshabur, B. *et al. The role of over-parametrization in generalization of neural networks* in *International Conference on Learning Representations* (2018).

3. Livni, R. *et al. On the computational efficiency of training neural networks* in *Advances in neural information processing systems* (2014), 855–863.

4. Arpit, D. *et al.* A closer look at memorization in deep networks. *arXiv preprint arXiv:1706.05394* (2017).

5. Gao, W. *et al.* Dropout Rademacher complexity of deep neural networks. *Science China Information Sciences* **59,** 072104 (2016).

6. Elsken, T. *et al.* Neural architecture search: A survey. *arXiv preprint arXiv:1808.05377* (2018).

7. *Automated Machine Learning: Methods, Systems, Challenges* (eds Hutter, F. *et al.*) In press, available at http://automl.org/book. (Springer, 2018).

8. Cortes, C. *et al. Adanet: Adaptive structural learning of artificial neural networks* in *International conference on machine learning* (2017), 874–883.

9. Liu, C. *et al. Progressive neural architecture search* in *Proceedings of the European Conference on Computer Vision (ECCV)* (2018), 19–34.

10. Zoph, B. *et al.* Neural architecture search with reinforcement learning. *arXiv preprint arXiv:1611.01578* (2016).

11. Liu, H. *et al.* Darts: Differentiable architecture search. *arXiv preprint arXiv:1806.09055* (2018).

12. Pan, S. J. *et al.* A survey on transfer learning. *IEEE Transactions on knowledge and data engineering* **22,** 1345–1359 (2010).

13. Acquarelli, J. *et al.* Convolutional neural networks for vibrational spectroscopic data analysis. *Analytica chimica acta* **954,** 22–31 (2017).

14. Zhang, X. *et al.* DeepSpectra: An end-to-end deep learning approach for quantitative spectral analysis. *Analytica chimica acta* **1058,** 48–57 (2019).

15. Ni, C. *et al.* Variable weighted convolutional neural network for the nitrogen content quantization of Masson pine seedling leaves with near-infrared spectroscopy. *Spectrochimica Acta Part A: Molecular and Biomolecular Spectroscopy* **209,** 32–39 (2019).

16. Malek, S. *et al.* One-dimensional convolutional neural networks for spectroscopic signal regression. *Journal of Chemometrics* **32,** e2977 (2018).

17. Liu, J. *et al.* Deep convolutional neural networks for Raman spectrum recognition: a unified solution. *Analyst* **142,** 4067–4074 (2017).

18. LeCun, Y. *et al.* Gradient-based learning applied to document recognition. *Proceedings of the IEEE* **86,** 2278–2324 (1998).

19. Lu, J. *et al.* Transfer learning using computational intelligence: A survey. *Knowledge-Based Systems* **80,** 14–23 (2015).

20. Kamaleswaran, R. *et al.* A robust deep convolutional neural network for the classification of abnormal cardiac rhythm using single lead electrocardiograms of variable length. *Physiological measurement* **39,** 035006 (2018).

21. Kessner, D. *et al.* ProteoWizard: open source software for rapid proteomics tools development. *Bioinformatics* **24,** 2534–2536 (2008).

22. Gatto, L. *et al.* MSnbase-an R/Bioconductor package for isobaric tagged mass spectrometry data visualization, processing and quantitation. *Bioinformatics* **28,** 288–289 (2012).

23. Santos, C. R. *et al.* Lipid metabolism in cancer. *The FEBS journal* **279,** 2610–2623 (2012).

24. Beloribi-Djefaflia, S. *et al.* Lipid metabolic reprogramming in cancer cells. *Oncogenesis* **5,** e189–e189 (2016).

25. Long, J. *et al.* Lipid metabolism and carcinogenesis, cancer development. *American journal of cancer research* **8,** 778 (2018).

26. Maan, M. *et al.* Lipid metabolism and lipophagy in cancer. *Biochemical and biophysical research communications* **504,** 582–589 (2018).

27. Cheng, C. *et al.* Lipid metabolism reprogramming and its potential targets in cancer. *Cancer Communications* **38,** 27 (2018).

28. Cameron, S. J. *et al.* Utilisation of Ambient Laser Desorption Ionisation Mass Spectrometry (ALDI-MS) Improves Lipid-Based Microbial Species Level Identification. *Scientific reports* **9,** 1–8 (2019).

29. Conrads, T. P. *et al.* High-resolution serum proteomic features for ovarian cancer detection. *Endocrine-related cancer* **11,** 163–178 (2004).

30. Thiele, H. *et al.* 2D and 3D MALDI-imaging: conceptual strategies for visualization and data mining. *Biochimica et Biophysica Acta (BBA)-Proteins and Proteomics* **1844,** 117–137 (2014).

31. Krizhevsky, A. *et al.* *Imagenet classification with deep convolutional neural networks* in *Advances in neural information processing systems* (2012), 1097–1105.

32. Chollet, F. *et al.* *Keras (2015)* 2017.

33. Pedregosa, F. *et al.* Scikit-learn: Machine learning in Python. *the Journal of machine Learning research* **12,** 2825–2830 (2011).
