# Supplementary material for: Cumulative learning enables convolutional neural network representations for small mass spectrometry data classification
Source: Nat Commun. 2020 Nov 5;11:5595. doi: 10.1038/s41467-020-19354-z (PMC7644674; doi:10.1038/s41467-020-19354-z)
Supplement: Supplementary file 3 — Description of Additional Supplementary Files [file 41467_2020_19354_MOESM3_ESM.pdf]

Description of Additional Supplementary Files

Title : Supplementary Data 1
Description : Correlation matrix with Pearson and cosine coefficients for the canine sarcoma dataset - healthy class

Title : Supplementary Data 2
Description : Correlation matrix with Pearson and cosine coefficients for the canine sarcoma dataset - Myxosarcoma class

Title : Supplementary Data 3
Description : Correlation matrix with Pearson and cosine coefficients for the canine sarcoma dataset – Fibrosarcoma class

Title : Supplementary Data 4
Description : Correlation matrix with Pearson and cosine coefficients for the canine sarcoma dataset – Hemangiopericytoma class

Title : Supplementary Data 5
Description : Correlation matrix with Pearson and cosine coefficients for the canine sarcoma dataset - Malignant peripheral nerve tumor class

Title : Supplementary Data 6
Description : Correlation matrix with Pearson and cosine coefficients for the canine sarcoma dataset – Osteosarcoma class

Title : Supplementary Data 7
Description : Correlation matrix with Pearson and cosine coefficients for the canine sarcoma dataset - Undifferentiated pleomorphic sarcoma class

Title : Supplementary Data 8
Description : Correlation matrix with Pearson and cosine coefficients for the canine sarcoma dataset - Rhabdomyosarcoma class

Title : Supplementary Data 9
Description : Correlation matrix with Pearson and cosine coefficients for the canine sarcoma dataset – Splenic fibrohistiocytic nodules class

Title : Supplementary Data 10
Description : Correlation matrix with Pearson and cosine coefficients for the canine sarcoma dataset - Histiocytic sarcoma class

Title : Supplementary Data 11
Description : Correlation matrix with Pearson and cosine coefficients for the canine sarcoma dataset -Soft tissu sarcoma class

Title : Supplementary Data 12
Description : Correlation matrix with Pearson and cosine coefficients for the canine sarcoma dataset - Gastrointestinal stromal sarcoma class

Title : Supplementary Data 13
Description : Correlation matrix with Pearson and cosine coefficients for the microorganism dataset
